# Reassessment of Species Diversity of the Subfamily Denticollinae (Coleoptera: Elateridae) through DNA Barcoding

**DOI:** 10.1371/journal.pone.0148602

**Published:** 2016-02-05

**Authors:** Taeman Han, Wonhoon Lee, Seunghwan Lee, In Gyun Park, Haechul Park

**Affiliations:** 1 Applied Entomology Division, Department of Agricultural Biology, National Academy of Agricultural Science, Wansan-gu, Jeonju, Korea; 2 Animal and Plant Quarantine Agency, Manan-gu, Anyang-si, Gyeonggi-do, Korea; 3 Division of Entomology, School of Agricultural Biotechnology, Seoul National University, Gwanak-gu, Seoul, Korea; Consiglio Nazionale delle Ricerche (CNR), ITALY

## Abstract

The subfamily Denticollinae is a taxonomically diverse group in the family Elateridae. Denticollinae includes many morphologically similar species and crop pests, as well as many undescribed species at each local fauna. To construct a rapid and reliable identification system for this subfamily, the effectiveness of molecular species identification was assessed based on 421 *cytochrome c oxidase subunit I* (*COI*) sequences of 84 morphologically identified species. Among the 84 morphospecies, molecular species identification of 60 species (71.4%) was consistent with their morphological identifications. Six cryptic and/or pseudocryptic species with large genetic divergence (>5%) were confirmed by their sympatric or allopatric distributions. However, 18 species, including a subspecies, had ambiguous genetic distances and shared overlapping intra- and interspecific genetic distances (range: 2.12%–3.67%) suggesting incomplete lineage sorting, introgression of mitochondrial genome, or affection by endosymbionts, such as *Wolbachia* infection, between species and simple genetic variation within species. In this study, we propose a conservative threshold of 3.6% for convenient molecular operational taxonomic unit (MOTU) identification in the subfamily Denticollinae based on the results of pairwise genetic distances analyses using neighbor-joining, mothur, Automatic Barcode Gap Discovery analysis, and tree-based species delimitation by Poisson Tree Processes analysis. Using the 3.6% threshold, we identified 87 MOTUs and found 8 MOTUs in the interval between 2.5% to 3.5%. Evaluation of MOTUs identified in this range requires integrative species delimitation, including review of morphological and ecological differences as well as sensitive genetic markers. From this study, we confirmed that *COI* sequence is useful for reassessing species diversity for polymorphic and polytypic species occurring in sympatric and allopatric distributions, and for a single species having an extensively large habitat.

## Introduction

Coleoptera is the most diverse order in the world with nearly 400,000 named species [1]. Many cryptic species have been reported in this order (e.g. [2–9]). Recognition of cryptic species diversity is essential to establishing conservation policies and pest control strategies for focal species. However, extremely similar or indistinguishable morphological features represented in many Coleoptera species have at times impaired morphological identification [10]. DNA barcoding has recently become entrenched as the standard method for molecular species identification [11], achieving successful identification rates of up to 97% in various animal taxa [12–16]. As a result, analysis of DNA sequences is currently regarded as essential for the detection of hidden species [17–18].

The family Elateridae, consisting of 13 subfamilies [19], is a large taxonomic group in the Coleoptera order and encompasses more than 10,000 described species worldwide [20–21]. In Elateridae, many new species are continually being identified and described; and taxonomic modifications are being made at the species level. Four DNA barcoding studies have been reported in this group, revealing the usefulness of DNA sequencing in making morphologically difficult or cryptic species identifications [22–25]. Oba et al. [22] adapted molecular identification and constructed phylogenetic relationships of the Japanese *Actenicerus* species (belonging to the subfamily Denticollinae) based on the *cytochrome c oxidase subunit I* (*COI*) and *28S rRNA* genes. Leseigneur et al. [23] also used *COI* to evaluate the taxonomic status of *Athous puncticollis* (Denticollinae), which had been considered synonymous with *Athous vittatus*, but confirmed by that study to be a distinct species. Staudacher et al. [24] applied *COI* analysis to identify the morphologically undetermined larvae of the genus *Agriotes* (subfamily: Elaterinae), which is a major crop pest. Furthermore, Wysockata et al. [25] revealed the possibility of hybridization between *Athous haemorrhoidalis* and *A*. *vittatus* based on their morphology and *COI* analysis.

Denticollinae Stein & Weise, 1877 is a cosmopolitan and morphologically diverse subfamily in Elateridae [26] and consists of 11 tribes (including Hypnoidinae), about 250 genera, and approximately 2,000 species worldwide [21, 27]. In this subfamily, many species belonging to the genera *Athous*, *Cidnopus*, *Drasterius*, *Limonius*, and *Selatosomus* are serious agricultural pests, causing damage to cultivated crops such as potato, wheat, sorghum, and corn [28–33]. Notably, a species of *Selatosomus* has often caused severe damage to potato fields in Korea [34–35]. Reassessment of morphologically identified species in this group by a molecular approach such as DNA barcoding is necessary i) to discover morphologically hidden species such as cryptic and pseudocryptic species [36–37] and ii) to uncover “oversplitting” (the misidentification of intraspecific variation as species-level variation) or “overlumping” (the misidentification of species-level variation as intraspecific variation) [38] in species that have extensive morphological variations across their geographical range.

In Korea, since the first faunistic report on 3 Denticollinae species by Heyden [39], 46 species have been identified in 19 faunistic reports and 9 taxonomic studies [30, 40–47]. In previous unpublished studies, we examined morphologies of the 46 species and found many erroneously recorded species, newly recorded species, and putatively new species in Korea. In this study, 391 *COI* sequences from 62 Denticollinae species collected in Korea and other countries from 2007 to 2013 were analyzed to provide more abundant taxonomic information to i) detect hidden species, ii) delimit species boundary in taxonomically difficult taxa represented in closely related species and within morphologically variable species, iii) confirm newly recorded and putatively new morphospecies supported by distinct monophyletic clustering, and iv) define genetically distinct intraspecific groups (haplotypes). Integrating morphological and molecular analyses can contribute to the construction of a more reliable species library than using a solely morphological approach. This combined approach can also provide an important foundation for rapid species assessment by accumulating sequence data for future global analyses of DNA barcoding. Our study was aimed to reassess morphologically identified species belonging to Denticollinae and to explore the minimum threshold value that should be applied to molecular species delimitation in the Denticollid taxa using the DNA barcode method.

## Materials and Methods

### Specimen collection and morphospecies identification

A total of 391 adult specimens from 62 species were collected in Korea (298 specimens of 36 species), Japan (41 specimens of 10 species), Russia (45 specimens of 14 species), and several other countries (6 specimens of 5 species), including Mongolia, Kyrgyzstan, Uzbekistan, and several European countries between 2001 and 2012. Most click beetles, the common name for species belonging to the Elateridae family, were hand-picked or captured in the field and either immediately placed in 100% ethanol (225 specimens) or kept alive until they could be stored at -80°C (76 specimens) for DNA extraction. Thirty-six specimens were obtained by using malaise traps with a cylinder containing 100% ethanol. Because other insects were trapped with our specimens and to avoid genetic contamination, we washed each specimen in running distilled water at least five times and preserved it at -80°C until genetic analysis could be performed. Fifty-three dried specimens were also used in *COI* analysis (**S1 Table**). No permits were required to collect the specimens in the field, and our institutional property rules were followed in all specimen collections.

The 390 specimens were identified based on morphology by the elaterid taxonomists Dr. T. Han (the first author in this study) for Korean and Russian species, Dr. H. Ôhira for Japanese species, and Dr. G. Platia for European species. In the process of species identification, we identified 2 new genera and 9 new species from the 62 morphospecies. The voucher specimens were stored in the insect specimen room at the Department of Agricultural Biology, National Academy of Agricultural Biology, Jeonju, Korea. The province abbreviations used in **S1 Table** and the figures are as follows: GW, Gangwon-do; GB, Gyeongsangbuk-do; GN, Gyeongsangnam-do; GG, Gyeonggi-do including Seoul; CB, Chungcheongbuk-do; CN, Chungcheongnam-do; JB, Jeollabuk-do; JN, Jeollanam-do; and JJ, Jeju-do Island.

### DNA extraction and DNA barcode analyses

Genomic DNA was extracted by using a QIAamp DNA Mini Kit (Qiagen, Hilden, Germany) according to the manufacturer’s instructions with one exception: tissues were not pulverized. To protect rare specimens from destructive DNA extraction, the following nondestructive method was used: the entire organism was incubated with rotation for 24 to 30 h in 0.8‒1.0 mL Buffer ATL, a tissue lysis buffer, which was increased in proportion to the organism’s body size, and 20‒80 μL proteinase K [37, 48]. Each DNA sample and its corresponding specimen were given the same sample identification number. After genomic DNA extraction, the specimens were repeatedly washed in distilled water and 100% ethyl alcohol. The washed, externally intact specimens were returned as dried specimens.

A 658 base pair (bp) *COI* sequence was amplified using the primer set LCO1490/HCO2198 [49]. But approximately one third of the samples were not amplified by this primer set. Therefore, we designed a specific primer set, suitable for Elateridae, based on a complete mitochondrial genome sequence (16,120 bp) of *Pyrophorus divergens* (from the subfamily Agrypninae) [50]. The primer set also accurately corresponds to the priming sites of LCO1490 and HCO2198 by Folmer *et al*. [49]: LCO1490Au (5'–TCAACAAACCATAAAGATATTGGAA–3') and HCO2198Au (5'–TAAACTTCTGGGTGTCCAAAGAATCA–3'). Polymerase chain reaction (PCR) amplifications were conducted with AccuPower PCR PreMix and HF PCR PreMix (Bioneer Daejeon, Korea) for 5 min at 94°C, followed by 35 cycles of 30 s at 94°C, 25 s at 50–52°C, and 1 min at 72°C; and a final extension for 5 min at 72°C. PCR products were assessed by 0.7% agarose gel electrophoresis, and successful amplicons were purified using a QIAquick PCR Purification Kit (Qiagen, Hilden, Germany). DNA sequencing was performed using an automated DNA analyzer (ABI 3730xl 96-capillary DNA analyzer; Applied Biosystems, USA) and the PCR primers. All products were sequenced from both strands. The quality and the possible polymorphic sites for the analyzed sequences were checked using Chromas 2.33 (Technelysium, Australia). In this step, we removed sequences with double or ambiguous peaks to avoid misleading signals in subsequent data analysis.

### Data analysis

Three hundred ninety-one *COI* sequences from the 62 species were successfully generated from the 391 samples. In addition, 30 *COI* sequences of 22 species published in five previous studies [11, 22, 24–25, 51] were downloaded from the GenBank (http://www.ncbi.nlm.nih.gov/genbank/). A combined dataset consisting of 421 sequences (the 391 sequences from the 62 collected species and the 30 sequences from the 22 GenBank species) was constructed (**S1 Table**). In accordance with the current classification by Cate [27], all sequences were taxonomically arranged corresponding to tribal, generic, and species groups as shown in **S1 Table**.

The data set of nucleotides was aligned in MEGA 5.2 [52] using ClustalW with the default settings (Gap Opening Penalty = 15, Gap Extension Penalty = 6.66 in both pairwise and multiple alignments). The anterior and posterior regions of uncertain alignment were eliminated from the data matrix. The *COI* alignment for reading frames was checked manually by translating sequences into amino acids to identify stop codons and potential shifts. All *COI* sequences were finally trimmed to 658 bp (**S1 File**). To avoid any misleading identifications by paralogous *COI* sequence co-amplifications such as nuclear mitochondrial pseudogenes (Numts) and heteroplasmy, we adopted the identification criteria of putative orthologues and paralogues in accordance with Moulton et al. [53] and Fontaneto et al. [54] for the sequences of conspecific individuals.

A neighbor-joining (NJ) analysis [55] was performed with MEGA 5.2. Genetic distances were calculated using Kimura’s 2-parameter test [56] in accordance with Nei’s empirical guidelines [57–58]. The NJ tree was represented using the online utility iTOL [59].

To estimate the number of molecular operational taxonomic units (MOTUs), each threshold value was calculated with mothur using the Hcluster command with the “Furthest neighbor” method [60]. We also examined two effective approaches to grouping hypothetical species, such as Automatic Barcode Gap Discovery (ABGD) based on pairwise genetic distances [61] and the Poisson Tree Processes (PTP) model based on the rooted phylogenetic trees [62]. ABGD automatically sorts the sequences into hypothetical species based on the barcoding gap by detecting pairwise distances between intra and interspecific variation from data and partitions the data accordingly. We used the ABGD web-server (http://wwwabi.snv.jussieu.fr/public/abgd/) to analyze our dataset. ABGD was run with the default settings (Pmin = 0.001, Pmax = 0.1, Steps = 10, Nb bins = 20), but two different values for relative gap width (*X* = 1.0, 1.5) were used with the Kimura K80 model. PTP is a tree-based species delimitation method using coalescence theory to distinguish between intra- and interspecies-level processes. This model assumes that intra- and interspecific substitutions follow two distinct Poisson processes and that intraspecific substitutions are significantly fewer than interspecific substitutions. The branch lengths should represent the number of substitutions [54, 62]. The rooted input-tree was constructed with RAxML [63] using the T-REX web-server [64] with the GTR+G+I substitution model.

## Results

### DNA barcoding of morphospecies

A total of 421 *COI* sequences representing 84 morphospecies belonging to 36 genera of 3 tribes, Hypnoidini, Denticollini, and Ctenicerini, were successfully generated to test the utility of DNA barcoding for species identification in the subfamily Denticollinae (**S2 Table**). Before analyzing the *COI* sequences, we confirmed the absence of any putative Numts and heteroplasmies from the 421 *COI* sequences.

Genetic distances in different taxonomic levels are shown in **Table 1**. The average interspecific genetic distance at the species level in each genus was 11.74% (range: 2.12%–27.70%). The average distance between genera within tribes was 19.76% (range: 8.40%–29.30%), and the average distance between tribes was 20.24% (range: 14.70%–32.30%). Among the 84 morphospecies, 46 morphospecies were represented by 2 or more specimens, whereas the other 38 species were represented by singletons.

**Table 1:** Genetic divergences in accordance with different taxonomic levels within Denticollinae.

Comparison	Number of comparisons	Average (%)	Minimum (%)	Maximum (%)	Standard error
Within species	3586	0.78	0.00	16.08	0.008
Within genus, between species	8575	11.74	2.12	27.70	0.060
Within tribes, between genera	23617	19.76	8.40	29.30	0.019
Between tribes	46861	20.24	14.70	32.30	0.017

Analyses of intraspecific genetic distances were carried out for the 46 morphospecies, and the average intraspecific distance was 0.78% (range: 0%–16.08%) (**Table 1**). Among the 46 morphospecies, 28 morphospecies showed low intraspecific distances, less than 2.0%; and another 13 species showed intraspecific distances ranging from 2.20% to 4.83%. The remaining five morphospecies revealed unexpectedly large maximum intraspecific distances of more than 5.0%: *A*. *vittatus* (14.97%), *Hemicrepidius oblongus* (5.13%), *Stenagostus umbratilis* (16.08%), *Selatosomus coreanus* (13.58%) and *Selatosomus koryeoensis* (7.76%). These large intraspecific distances suggested potential cryptic species in the five morphospecies (**S2 Table**).

Of the 3,486 species pairs from the 84 morphospecies analyzed using Kimura’s 2-parameter pairwise comparison, 3,479 species pairs revealed congeneric interspecific genetic distances ranging from 4.16% to 27.70%, indicating well defined species with a distinct genetic gap known as the “barcoding gap” [65]. However, the remaining 7 pairs consisting of 11 morphospecies belonging to 3 genera (*Actenicerus*, *Hemicrepidius*, and Ctenicerini gen. and sp. 1 and sp. 2) had low interspecific distances and ambiguously stretched genetic intervals (range: 2.12%–5.07%) (**Table 2**). Among these 7 morphospecies pairs, morphological differentiation between Ctenicerini gen. and sp. 1 and sp. 2 was well defined; however, members of the remaining pairs shared similar morphological characteristics. The lowest interspecific distance, 2.12%, was detected between Ctenicerini gen. and sp. 1–C. sp. 2 (range: 2.12%–3.54%) and the second lowest between *Hemicrepidius* sp. 1 and *H*. *oblongus* (range: 2.74%–3.38%). The other five pairs exhibited interspecific distances ranging from 3.22% to 5.07%. When disregarding the five cryptic species complexes (*A*. *vittatus*, *H*. *oblongus*, *S*. *umbratilis*, *S*. *coreanus*, and *S*. *koryeoensis*) (**S3 Table**), intra- and interspecific genetic distances overlapped in some species. For example, the pair Ctenicerini gen. and sp. 1–C. sp. 2 had a minimum interspecific distance of 2.12%, whereas *Prosternon aurichalceum* had a maximum intraspecific divergence of 4.82%.

**Table 2:** The seven congeneric morphospecies pairs with low interspecific distances.

Species pairs	Morphological difference	Range of interspecific genetic distance	Number of MOTUs	
			2.5% threshold	3.5% threshold
Ctenicerini gen. and sp. 1 (11)/Ctenicerini gen. and sp. 2 (3)	Distinct	2.12–3.54%	3	1
*Actenicerus kidonoi* (1)/*A*. *giganteus* (1)	similar	3.35%	2	1
*Actenicerus orientalis* (1)/*A*. *naomii* (1)*	similar	3.69%	2	2
*Hemicrepidius* sp. 1 (1)/*H*. *oblongus* (20)	similar	2.74–3.38%	2	1
*Hemicrepidius* sp. 1 (1)/ the Jeju population of *H*. *coreanus* (57)*	similar	3.22–3.86%	2	2
*Hemicrepidius* sp. 2 (7)/ *H*. *hallaensis* (10)*	similar	3.23–4.32%	2	2
*Hemicrepidius* sp. 2 (7)/ the Korean population of *H*. *oblongus* (18)*	similar	3.55–5.07%	2	2

Parenthetical numerals denote the number of examined specimens.

* denotes an independent MOTU taxa applying the 3.6% threshold proposed in this study.

### MOTUs estimation

**Fig 1:**
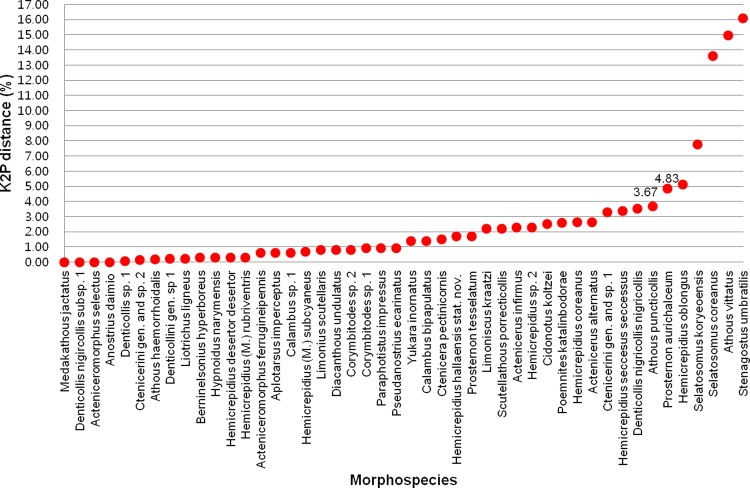
Maximum intraspecific distances by Kimura-2-parameter (K2P) for 46 morphospecies.

To investigate the appropriate threshold value for evaluating the number of MOTUs within the Denticollid taxa, using mothur [60], we examined the maximum intraspecific distance within each of the 46 morphospecies, which included multiple samples. We found a clear gap of 3.67% in *A*. *puncticollis* and another of 4.83% in *P*. *aurichalceum* (**Fig 1**). *A*. *puncticollis* had two genetic groups showing lower intragroup distances (range: 0–1.16%) than intergroup distances (range: 1.50%–3.67%). *P*. *aurichalceum* had three genetic groups with lower intragroup distances (range: 0.30%–2.12%) than intergroup distances (range: 3.37%–4.83%) (**S3 Table**). The genetic distances between the subgroups in the two species were similar to interspecific distances of four congeneric species pairs (*Actenicerus orientalis*–*A*. *naomii*, *Hemicrepidius* sp. 1–the Jeju population of *H*. *coreanus*, *H*. sp. 2–*H*. *hallaensis*, and *H*. sp. 2–the Korean population of *H*. *oblongus*), which ranged from 3.22% to 4.32% (**Table 2, S3 Table**). From the 84 morphospecies, we concluded a 3.6% threshold value is reasonable to delineate Denticollid species. This threshold was suggested by previous studies [66–68] (**Fig 1**). Based on the 3.6% threshold, 87 MOTUs were identified by adding to the original 84 morphospecies the 7 newly identified lineages hidden in 7 morphospecies (*A*. *puncticollis*, *A*. *vittatus*, *P*. *aurichalceum*, *H*. *oblongus*, *S*. *koryeoensis*, *S*. *coreanus*, and *S*. *umbratilis*) with a large intergroup genetic distance (range: 3.67%–16.08%), and excluding 4 species from the 4 species pairs (Ctenicerini gen. and sp. 1–sp. 2, *A*. *kidonoi*–*A*. *giganteus*, *H*. sp. 1–*H*. *oblongus*, and *H*. sp. 1–the Russian population (no. 2397) of *H*. *oblongus*) with an interspecific distance less than 3.6% (**Table 2**). However, this threshold did not appear to be suitable for application to our entire dataset because some distinct morphospecies (e.g., Ctenicerini gen. and sp. 1–C. sp. 2 and *A*. *kidonoi*–*A*. *giganteus*) were regarded as a single species using the 3.6% threshold.

**Fig 2:**
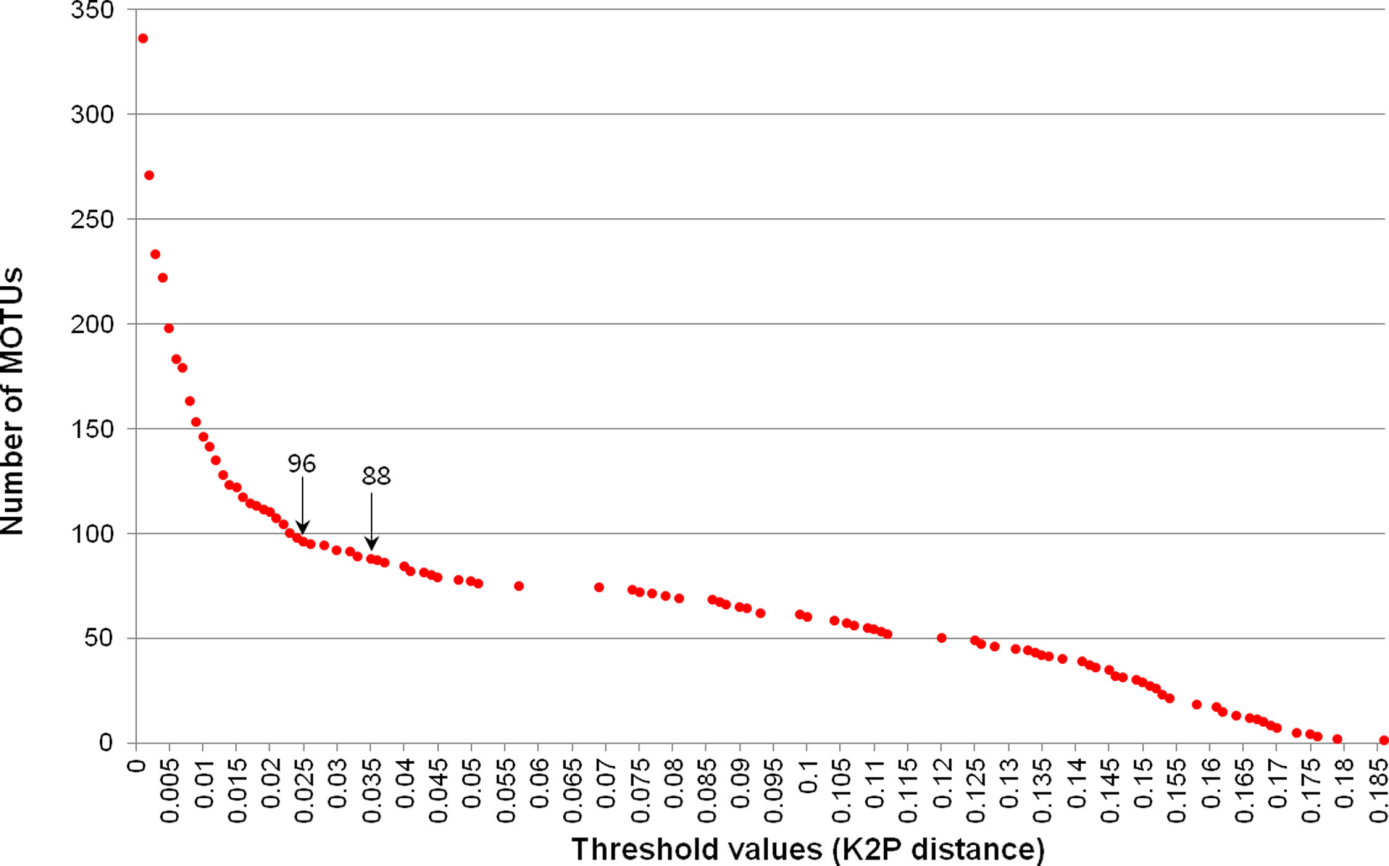
Number of MOTUs in accordance with different threshold values.

In the next step, we examined the number of MOTUs estimated by different threshold values based on our dataset and found an initial plateau at 2.5% (96 MOTUs) and a second at 3.5% (88 MOTUs), which were relatively insensitive to changes in the cut-off value, with a slow decline after 4.0% (**Fig 2**). These plateaux were suggested to sign as the most of the MOTUs are also a good biological species [66–68]. The 8 MOTUs created by decreasing the threshold from 3.5% to 2.5% involved three congeneric morphospecies pairs (Ctenicerini gen. and sp. 1–C. sp. 2, *A*. *kidonoi*–*A*. *giganteus*, and *H*. sp. 1–*H*. *oblongus*) (**Table 2**) and five morphospecies (*A*. *pucticollis*, *Hemicrepidus seccessus*, Ctenicerini gen. and sp. 1, *Actenicerus alternatus*, and *S*. *coreanus*). Notably, Ctenicerini gen. and sp. 1 had a single MOTU at both the 2.5% and 3.5% thresholds (**Table 3**). Among these species, only two showed one amino acid substitution in each of their sequence examinations. For example, 2 samples (nos. 2890 and 3113) out of the 11 specimens in Ctenicerini gen. and sp. 1 showed a point mutation (G**T**A → G**C**A at the 515 bp position); however, this had no effect on MOTU clustering. In addition, two samples (nos. 3077 and 3078) out of the ten specimens in *H*. *seccessus* showed two nucleotide substitutions, including one nonsynonymous substitution at 19 bp (first codon position) changing the leucine codon (**T**T**A**) to the isoleucine codon (**A**T**C**) and one silent substitution at 21 bp (third codon position). These substitution sites functioned as 2 parsimony informative sites and contributed to the formation of separate MOTUs. The remaining 6 morphospecies and morphospecies pairs had synonymous nucleotide substitutions in all sequences. Three congeneric species pairs (Ctenicerini gen. and sp. 1–C. sp. 2, *H*. sp. 1–*H*. *oblongus*, and *A*. *giganteus*–*A*. *kidonoi*) with members that were not genetically distinct using the 2.5% threshold could also be distinguished from each other by their morphological differences. However, the genetically distinct subgroups represented in 4 morphospecies (*A*. *alternatus*, *H*. *seccessus*, *P*. *katalinbodorae*, and *S*. *coreanus*) based on MOTUs identified with the 2.5% threshold were morphologically indistinguishable. Although *A*. *puncticollis* had 2 MOTUs at the 2.5% threshold, it was not examined morphologically in this study.

**Table 3:** The 11 morphospecies representing typically variable intraspecific distances.

Species	Maximum intraspecific genetic distance	Number of MOTUs	
		2.5% threshold	3.5% threshold
Athous pucticollis*	~3.67%	2	2
*Hemicrepidius* sp. 2	~2.29%	1	1
*Hemicrepidius oblongus*	~3.66%	1	1
*Hemicrepidius coreanus*	~2.62%	1	1
*Hemicrepidius seccessus*	~3.37%	2	1
Ctenicerini gen. and sp. 1	~3.31%	1	1
*Poemnites katalinbodorae*	~2.59%	2	1
*Actenicerus infirmus*	~2.28%	1	1
*Actenicerus alternatus*	~2.62%	2	1
within *Selatosomus coreanus*			
*S*. *coreanus* (?)	~3.23%	1	1
*S*. *reichardti* (?)	~3.5%	2	1

* denotes an independent MOTU taxa applying the 3.6% threshold determined in this study.

Question mark indicates uncertain species identification in this study.

**Fig 3:**
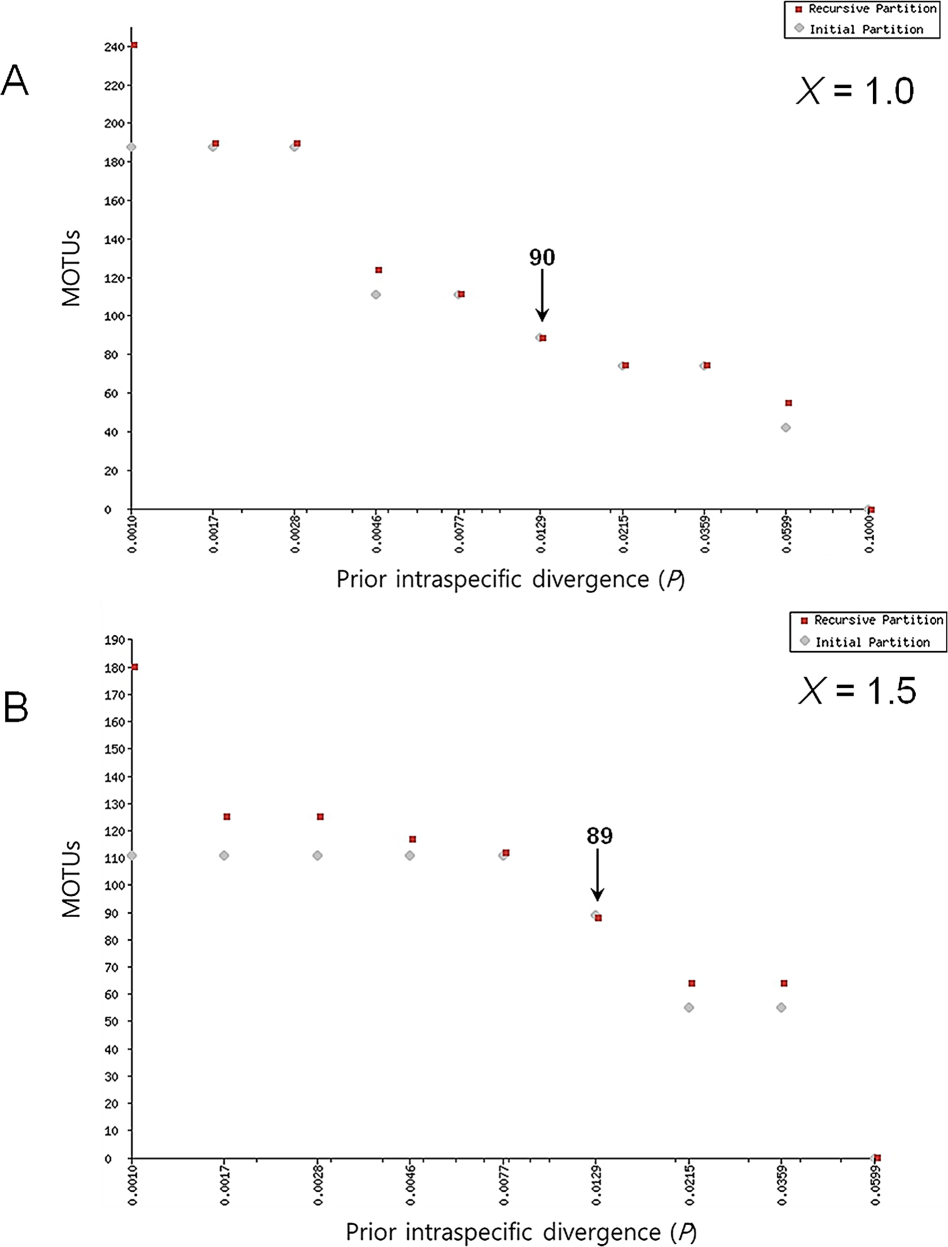
The number of MOTUs by the prior intraspecific divergence using ABGD with two values of relative gap width (*X* = 1.0 in A, 1.5 in B).

ABGD was used with its default settings, but we employed two values of relative gap width (*X* = 1.0, 1.5). The values of *X* produced different MOTU counts ranging from 56 to 242 when *X* = 1.0 and from 65 to 181 when *X* = 1.5, with variation resulting from the consistent value of prior intraspecific divergence (*P*) per each partition (**Fig 3A and 3B**). The number of MOTUs was 90 and 89 at *X* = 1.0 and *X* = 1.5, respectively, when *P* = 0.0129. These results were similar to the number of divergent mothur clusters (87 MOTUs based on the 3.6% threshold and 88 MOTUs by the 3.5% threshold). However, the ABGD analysis of MOTUs yielded several different results: *A*. *puncticollis* consisted of a unique cluster, and *A*. *kidonoi* and *A*. *giganteus* were independent species. The only discrepancy in species recognition occurred in *Yukara inornatus* with 2 MOTUs when *X* = 1.0 and 1 MOTU when *X* = 1.5, represented by a distinct haplotype (no. 2605) with 1.4% intraspecific genetic distance (**Table 4, S2 Table**).

**Table 4:** MOTU recognition per morphospecies using the ABGD method.

Morphosspecies	intraspecific genetic distances	interspecific genetic distances	MOTUs (*P* = 0.0129)	
			*X* = 1.0	*X* = 1.5
*A*. *puncticollis*(4)	0–3.67%		1	1
*A*. *vittatus* (4)	0–14.97%		2	2
*Hemicrepidius* sp. 1 (1)	–	2.47–5.13%	1	1
*H*. *oblongus* (20)	1.66–3.66%			
*H*. *oblongus*, Russia (no. 2397) (1)	–		1	1
*Y*. *inornatus* (20)	0–1.40%		2	1
*S*. *umbratilis* (16)	0–16.08%		2	2
*Denticollis nigricollis nigricollis* (15)	0–3.54%	2.12–3.54%	1	1
*D*. *nigircollis* subsp. 1 (3)	0			
Ctenicerini gen. and sp. 1 (11)	0–3.31%	2.12–3.54%	1	1
Ctenicerini gen. and sp. 2 (3)	0–0.15%			
*A*. *kidonoi* (1)/*A*. *giganteus* (1)		3.35%	2	2
*A*. *orientalis* (1)/*A*. *naomii* (1)		3.69%	2	2
*S*. *coreanus* (42)	0–13.58%		2	2
*S*. *koryeoensis* (8)	0–7.76%		2	2
*P*. *aurichalceum* (7)	0.20–4.82%		3	3

Parenthetical numerals denote the number of examined specimens. *X* is relative gap width.

*P* is prior intraspecific divergence.

PTP analysis yielded 101 MOTUs with the maximum likelihood solution (**S2 File**). This result was closer to the 96 MOTUs we identified using a 2.5% threshold than the 87 MOTUs we identified using a 3.6% threshold in mothur, but PTP analysis more sensitively delimitated the species. For example, 3 morphological species (*Cidonotus koltzei*, *H*. *hallaensis* and *Paraphotistus impressus*) had 2 MOTUs each using PTP analysis, even though the maximum intraspecific distances of these species were significantly low, ranging from 0.9% to 2.50% (**Table 5**). This overestimation of species may have been due to an insufficient number of samples or the presence of small evolutionary substitutions within a species [62].

**Table 5:** MOTU recognition per morphospecies in the Poisson Tree Processes model.

Morphosspecies	No. of individuals	Intraspecific genetic distances	Interspecific genetic distances	MOTUs	Status
*C*. *koltzei*	2	2.50%		2	splitting
*A*. *puncticollis*	4	0–3.67%		3	splitting
*A*. *vittatus*	4	0–14.97%		3	splitting
*H*. *hallaensis*	10	0–1.70%		2	splitting
*H*. *oblongus*	21	0–5.13%		2	splitting
*H*. *seccesus seccessus*	10	0–3.37%		2	splitting
*S*. *umbratilis*	16	0–16.08%		2	splitting
*D*. *nigricollis nigricollis*	15	0–3.54%		1	sharing
*D*. *nigircollis* subsp. 1	3	0			
Ctenicerini gen. and sp. 1	11	0–3.31%	2.12–3.54%	1	sharing
Ctenicerini gen. and sp. 2	3	0–0.15%			
*Poemnites katalinbodorae*	5	0.20–2.59%		3	splitting
*A*. *alternatus*	3	0–2.62%		2	splitting
*A*. *kidonoi*	1	–	3.35%	1	splitting
*A*. *kiashianus*	1	–		1	splitting
*S*. *coreanus*	42	0–13.58%		2	splitting
*S*. *koryeoensis*	8	0–7.76%		2	splitting
*P*. *impressus*	2	0.9%		2	splitting
*P*. *aurichalceum*	7	0.20–4.82%		4	splitting

From the species delimitations based on morphology, DNA barcode approaches comparing pairwise genetic distances, such as traditional DNA barcoding [12] and ABGD [61], and tree-based approaches, such as PTP [62], our results suggested that many Denticollid species had undergone random speciation and that the related morphological change is faster than *COI* gene divergence in some instances and slower in others for genetic distances between 2.5% to 3.5%. Therefore, the 3.6% threshold showed congruence with both morphological and molecular taxonomic unit separation in our dataset and appears to be a suitable threshold for the Denticollid taxa. However, an integrative taxonomic approach may have to be applied to any subsequently identified species with an intraspecific genetic distance between 2.5% to 3.5% for more precise species recognition [69].

### DNA barcoding tree and cases of species delimitation

**Fig 4:**
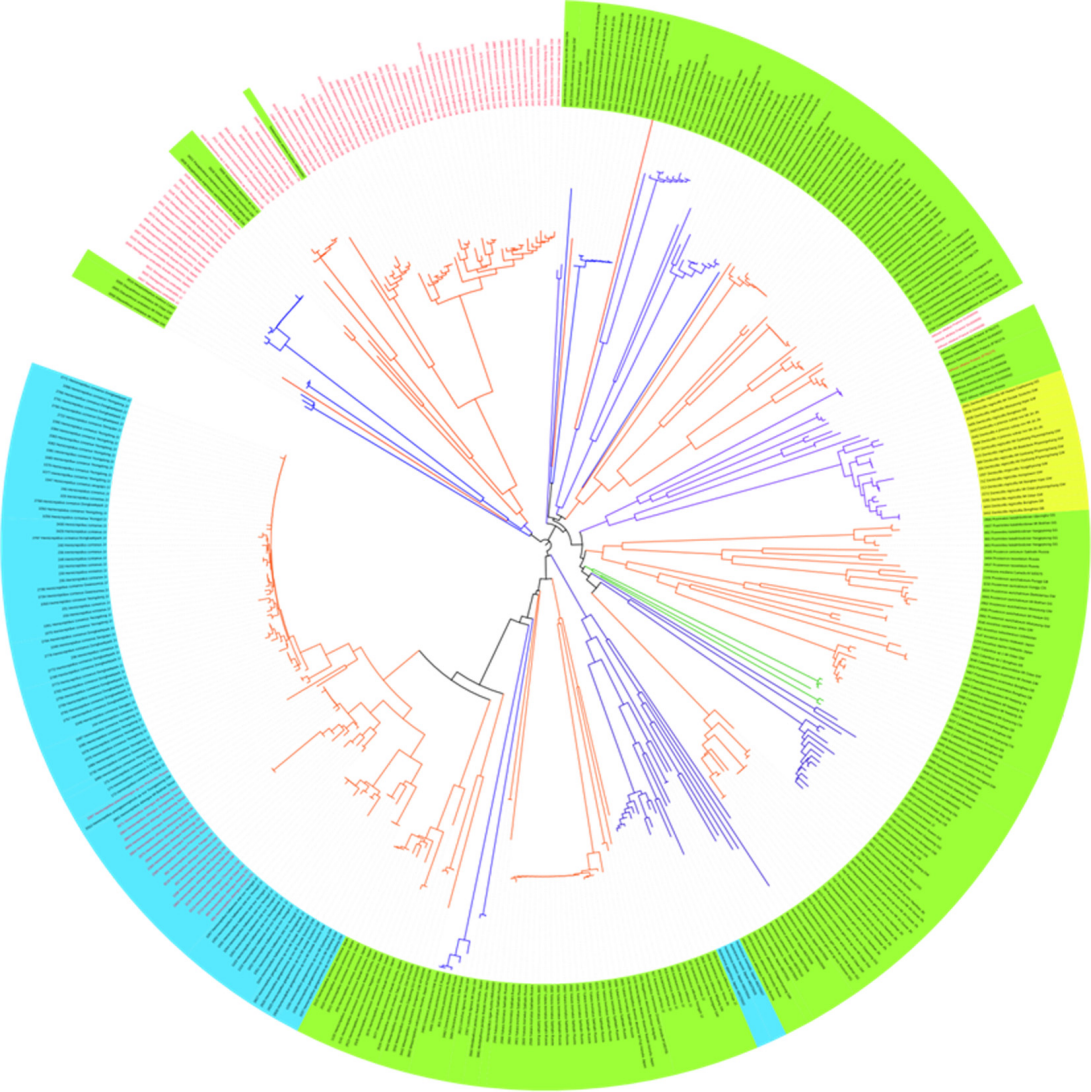
Neighbor-joining tree for the 420 individuals of the 84 morphospecies in this study based on *COI* barcode sequences. Clade colors represent three different tribes: the green clades denote Hypnoidini, the red clades represent Ctenicerini, and the blue clades show Denticollini. The red text indicates the unexpectedly highly divergent taxa in a single species. The blue highlight denotes unexpectedly low divergence between a species pair, the green highlight represents the taxa successfully identified by DNA barcode, and the yellow highlight indicates ambiguously defined subspecies.

The NJ tree profile (**Fig 4**) showed that sequence records for 60 (71.4%) of the 84 morphospecies form distinct species clusters with unambiguous identification when correlated with our prior morphological study. Analysis of the remaining 24 morphospecies revealed i) 6 cryptic and/or pseudocryptic species from 6 morphospecies, ii) ambiguous genetic distances in the 2.12% to 3.67% range within and between 15 species thought to be caused by incomplete lineage sorting and simple genetic variation in each species, and iii) DNA barcode sharing between morphologically distinct species (2 spp.: Ctenicerini gen. and sp. 1–C. sp. 2) and subspecies (1 sp.: *Denticollis nigricollis nigricollis*–*D*. *n*. subsp. 1). These are explained separately in seven cases as follows.

#### 1) Discovering sympatric cryptic species

**Fig 5:**
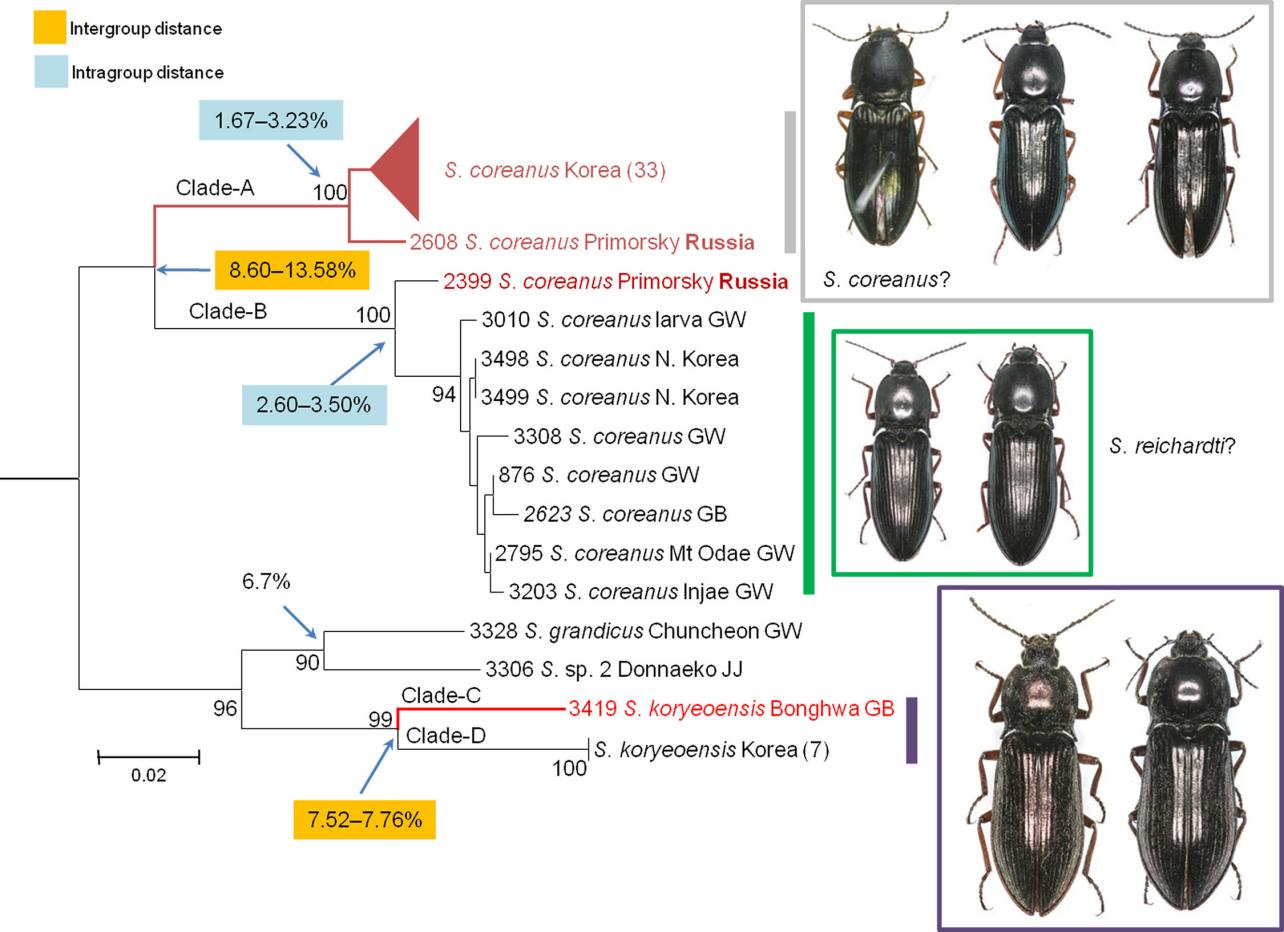
Neighbor-joining cladogram inferred from *COI* partial gene sequences of *Selatosomus* spp.

Four species of *Selatosomus* were analyzed in this study. DNA barcoding revealed two sympatric cryptic species from each of two morphospecies, *S*. *coreanus* and *S*. *koryeoensis* (**Fig 5**). In *S*. *coreanus*, 43 specimens formed two distinct clusters in all our analyses (NJ tree; mothur: **Table 3**; ABGD: **Table 4**; PTP: **Table 5**). Clade-A was considered as *S*. *coreanus* and consisted of 33 Korean specimens and 1 Russian specimen (no. 2608). Clade-A showed intraspecific divergences ranging from 1.67% to 3.23%. Clade-B consisted of nine specimens, including two North Korean specimens and one Russian specimen, showing intraspecific divergences ranging from 2.60% to 3.50%. The genetic divergences between Clade-A and Clade-B were significantly large, ranging from 8.60% to 13.58%. Morphologically, *S*. *coreanus* has variable coloration in the dorsal parts and legs; however, we found no distinct morphological characteristics distinguishing between the two clades.

Notably, several elaterid specialists have confused *S*. *coreanus* with *Selastomus reichardti*. *S*. *coreanus* was identified from organisms in Korea [40], and *S*. *reichardti* was identified from organisms in the Russian Far East [70]. However, the location of the type specimens of these 2 species is unknown [41]. Furthermore, *S*. *reichardti* was synonymized with *S*. *coreanus* by Kishii [41, 71] based on an examination of each topotype. Since then, the geographic distribution of the species has been found to extend further throughout Korea, North China, Mongolia, and the Russian Far East. Our study included the two Far East Russian specimens considered topotypes of *S*. *reichardti*. However, our result unequivocally revealed that two genetically divergent species exist sympatrically across Korea and the Russian Far East and are considered cryptic species. From this finding, we question whether the type specimens used in the original descriptions of *S*. *coreanus* and *S*. *reichardti* were the same species? If they are same species, one of the two clades identified in our analyses may be new to science and the other clade is *S*. *coreanus*.

Analyses of *S*. *koryeoensis* revealed another cryptic species. In *S*. *koryeoensis*, Clade-D consisted of seven individuals without intraspecific genetic divergence. Clade-C consisted of a single specimen (no. 3419) (**Fig 5**) with no morphological characteristics different from those of Clade-D members; but the genetic divergence between Clade-C and Clade-D was large, ranging from 7.52% to 7.76%. This divergence was not associated with other closely related congeneric species identified in the previous taxonomic revision [72].

#### 2) Discovering sympatric pseudocryptic species

**Fig 6:**
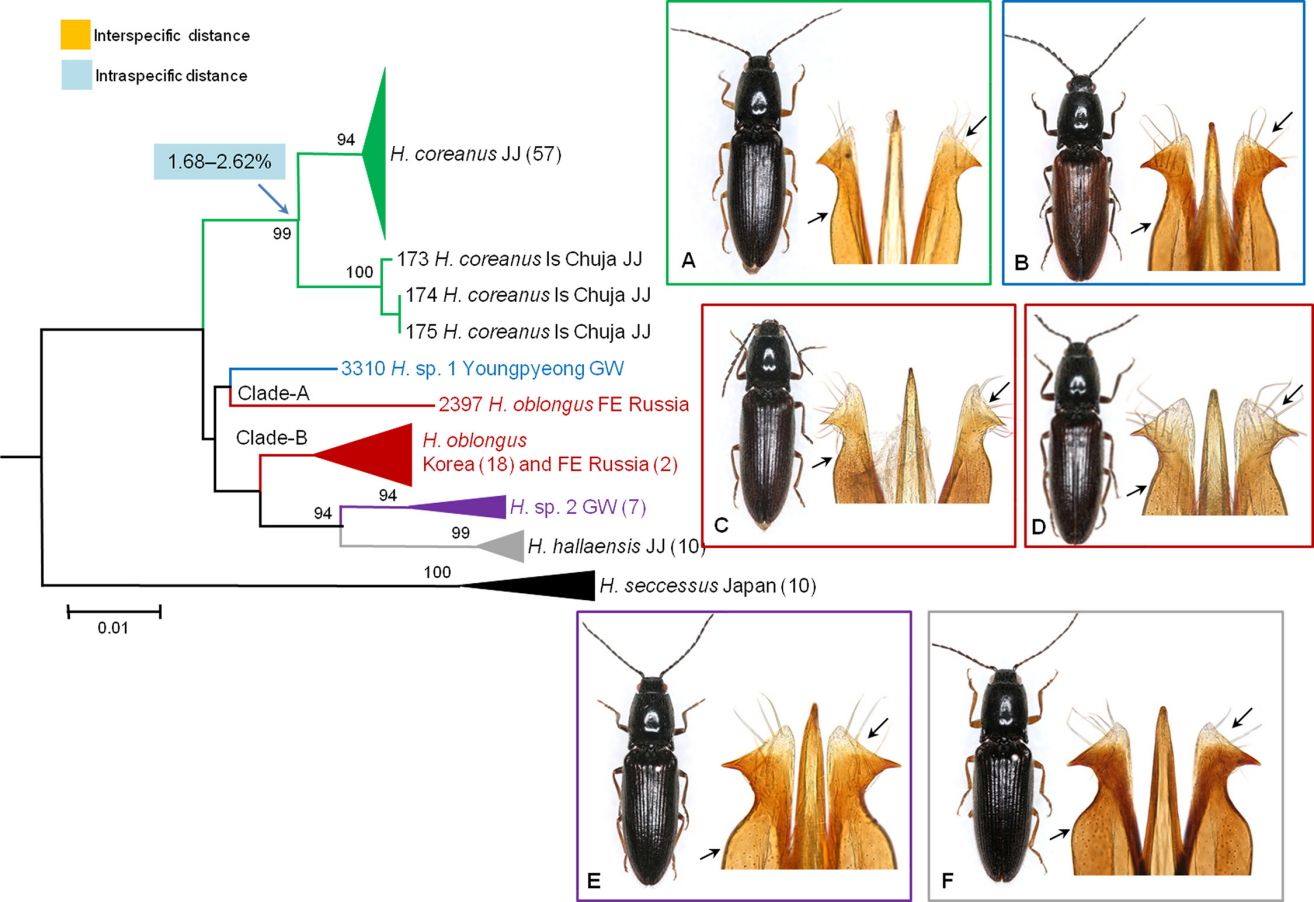
Neighbor-joining cladogram inferred from *COI* partial gene sequences of *Hemicrepidius* spp. and their habitus and aedeagus. A: *H*. *coreanus*; B. *H*. sp. 1 (no. 3310, a candidate new species); C: *H*. *oblongus* from Far East Russia (no. 2397, a candidate new pseudocryptic species); D: *H*. *oblongus*, Korea and Far East Russia; E. *H*. sp. 2, GW (a candidate new species); F: *H*. *hallaensis*, JJ.

In *H*. *oblongus* (**Fig 6**), Clade-A consisted of a single Russian specimen (no. 2397; **Fig 6C**). This specimen was identified as *H*. *oblongus* by sharing certain external features and collection site with the two other Russian specimens (nos. 2395 and 2396); however, the genetic distances between specimen no. 2397 and members of Clade-B were large, ranging from 4.16% to 5.13%. Specimen no. 2397 was reexamined morphologically and determined to have distinct morphological characteristics, the shape of the aedeagal parameres, which suggested that the specimen is a sympatric pseudocryptic species, rather than a cryptic species.

**Fig 7:**
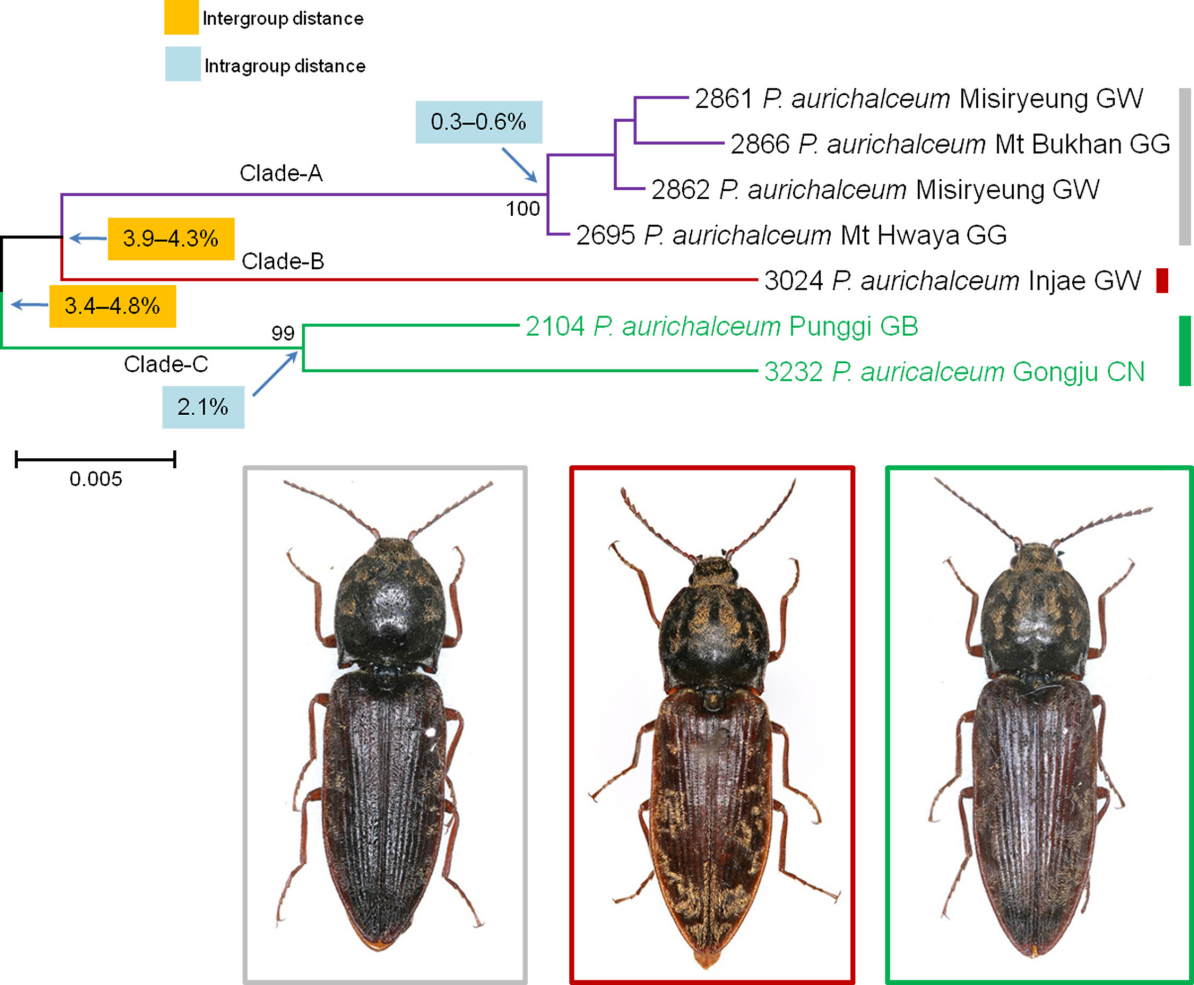
Neighbor-joining cladogram inferred from *COI* partial gene sequences of *Prosternon aurichalceum*.

We collected seven specimens of *P*. *aurichalceum* having seven different haplotypes with various intraspecific distances, ranging from 0.2% to 4.83% (**Fig 7**). According to the NJ tree, mothur, and ABGD, this group of specimens had three subgroups; but PTP analysis revealed four subgroups (**Table 5, S2 File**). The genetic distances within Clade-A and Clade-B were 0.3% to 0.6% and 2.1%, respectively, while the genetic distances between these two clades ranged from 3.9% to 4.3%. The genetic distances between Clade-A+B and Clade-C ranged from 3.4% to 4.8% (**Fig 7**). Subsequent morphological re-examination of the specimens revealed that the members of Clade-A had blackish bodies, whereas three specimens of Clade-B and Clade-C had paler bodies. The shapes of the aedeagus in Clade-A (no. 2695) and Clade-B (no. 3024) were indistinguishable from one another, but the shape of the aedeagus in Clade-C (no. 3232) was subtly different. These cases demonstrate the utility of DNA barcode analysis in recognizing pseudocryptic species overlooked in morphological species delimitation in traditional taxonomy.

**Fig 8:**
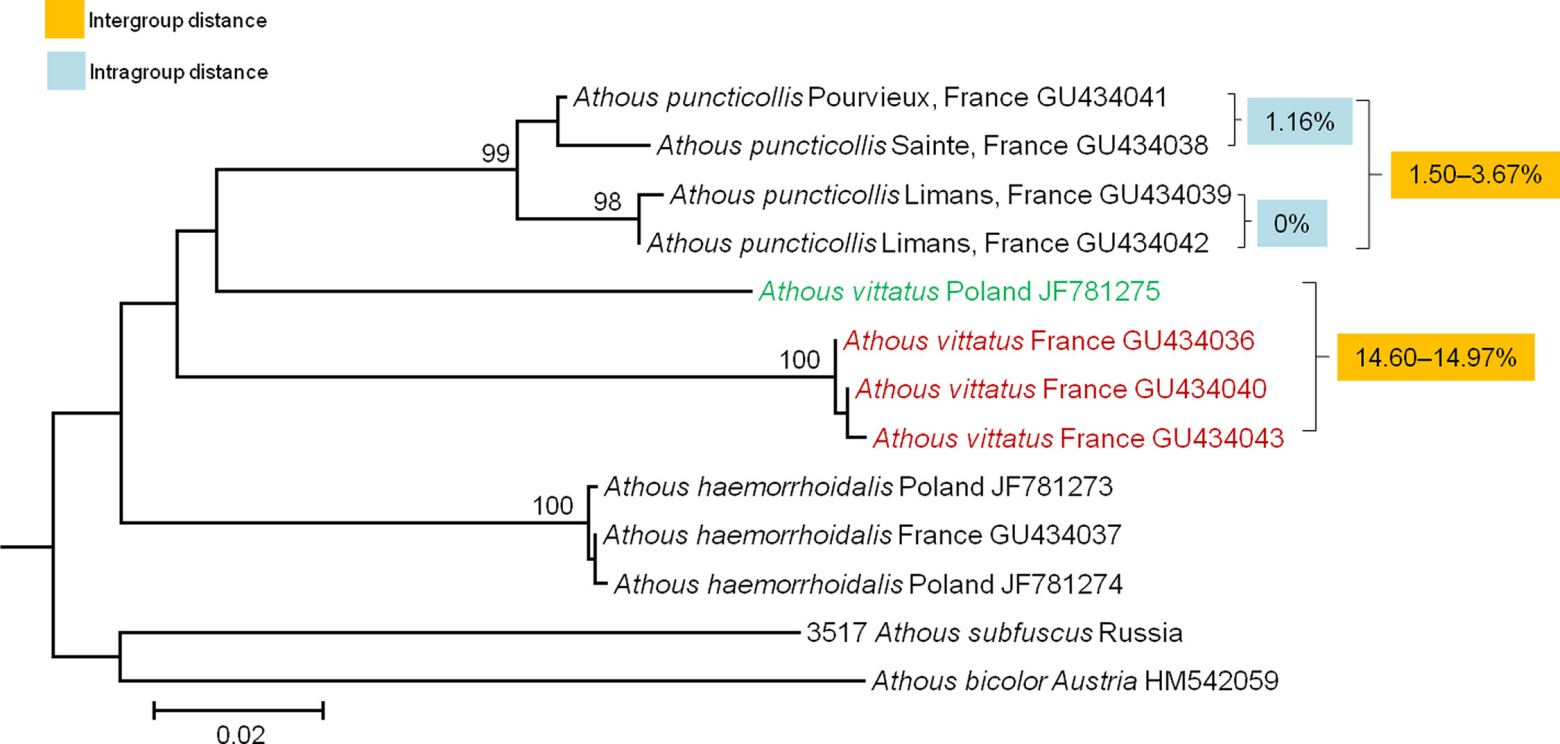
Neighbor-joining cladogram inferred from *COI* partial gene sequences of *Athous* spp.

*A*. *vittatus* is a common European click beetle that lives in a wide area extending from European countries to Turkey and has various forms [31, 51]. This species includes 25 synonymized species listed in “Catalogue of Palaearctic Coleoptera” [27]. Four *COI* sequences extracted from two previous studies [23, 25] showed large genetic distances (range: 14.60%–14.97%, **S3 Table**; **Fig 8**) between French and Polish populations. This is another example of cryptic species in Denticollinae [25].

#### 3) Discovering allopatric pseudocryptic species

**Fig 9:**
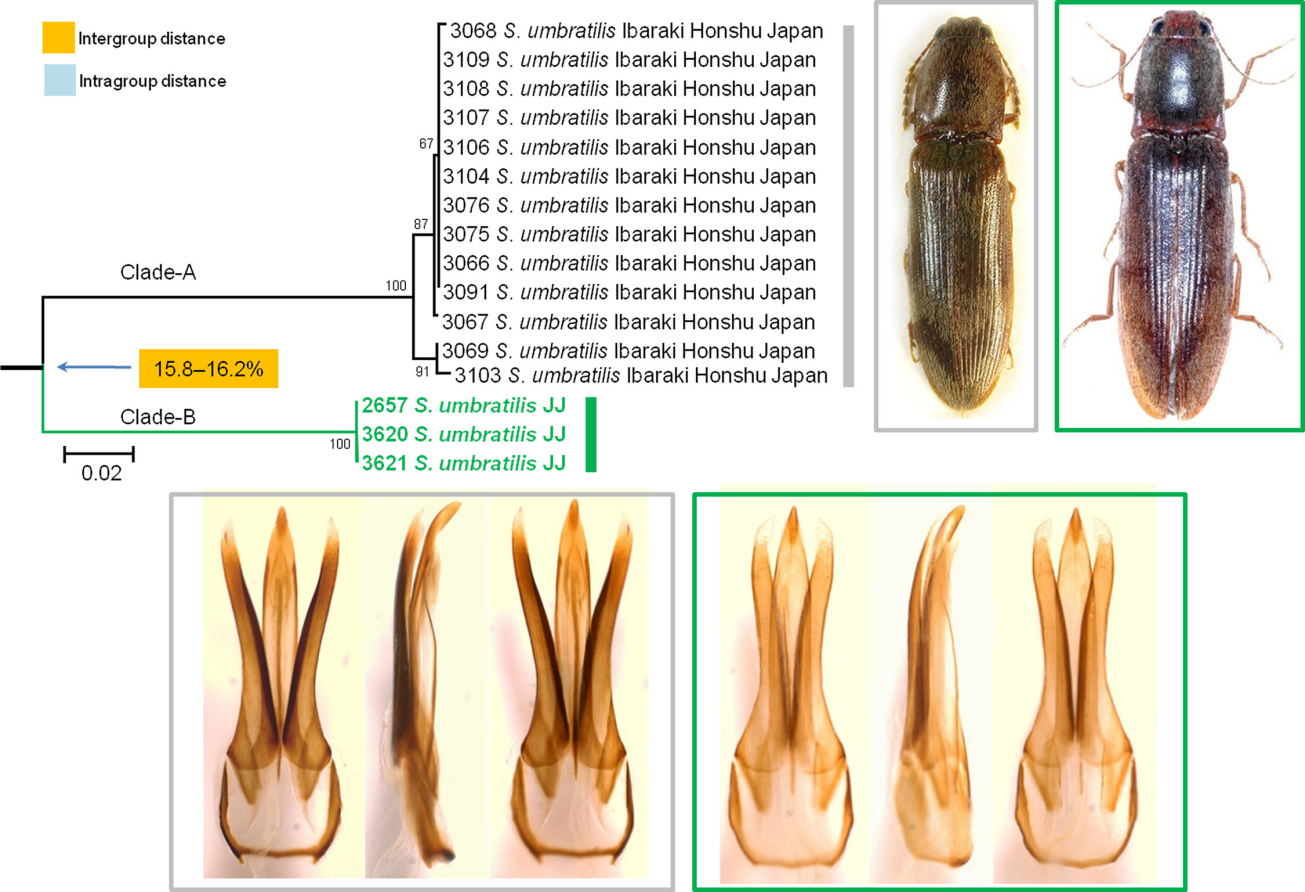
Neighbor-joining cladogram inferred from *COI* partial gene sequences of *Stenagostus umbratilis*.

*S*. *umbratilis* was originally described from Japan. The Korean population was first reported by Kim & Chang [73], and then the Jeju population was recorded by Kishii & Paik [42]. From our morphological study, we observed that the Jeju population was very similar to the Japanese population in appearance and the shape of the aedeagus, but the Jeju population was distinguished by a slightly longer antennae, half of the 11th antennomere extending to the apex of the pronotal hind angles in the male and barely reaching to the apex in the female, and more thickly pointed aedeagal parameres (**Fig 9**). Initially, confusion existed over whether these differentiations represented geographical individual variations, subspecies, or distinct species; but the genetic distance between these 2 populations was unexpectedly large. The subtle morphological differences combined with the highly divergent lineage should be sufficient evidence for establishing the Jeju population as a new pseudocryptic species; however, further study is needed considering the extremely larger genetic distance shown in this species pair compared with the typical interspecific distances between congeneric species.

#### 4) Ambiguous genetic distances cause difficulties in determining intra- and interspecific boundaries: how to solve this phenomenon?

Several morphospecies pairs and morphospecies had ambiguous genetic distances between and within species, respectively. Seven morphospecies pairs had relatively low interspecific distances (range: 2.12%–5.07%, expressed by stretched genetic distances within 1.6% intervals in each pair) (**Table 2**). Eleven morphospecies had relatively large maximum intraspecific distances (range: 2.28%–3.67%; 1.39% interval) (**Table 3**). The overlapping genetic distances between intra- and interspecific comparisons ranged from 2.12% to 3.67%. However, our suggested threshold of 3.6% could separate four species pairs, that included seven distinct species, with ambiguous species-level delimitation based on independent MOTUs (see asterisk marked taxa in **Table 2**). These species could also be distinguished by their subtly different morphological features and by ABGD and PTP analyses. When we applied the 3.6% threshold, the number of MOTUs for the 11 species listed in **Table 3**did not change. We found no consistent morphological differences within each species despite careful scrutiny for variable coloration or other subtle differences, especially for 3 species *H*. *oblongus*, *H*. *coreanus* and *H*. *seccessus* and the two divergent cryptic lineages of *S*. *coreanus*. The results of all our analyses indicate that both morphological and genetic variations represented in each of these species may be intraspecific variations.

**Fig 10:**
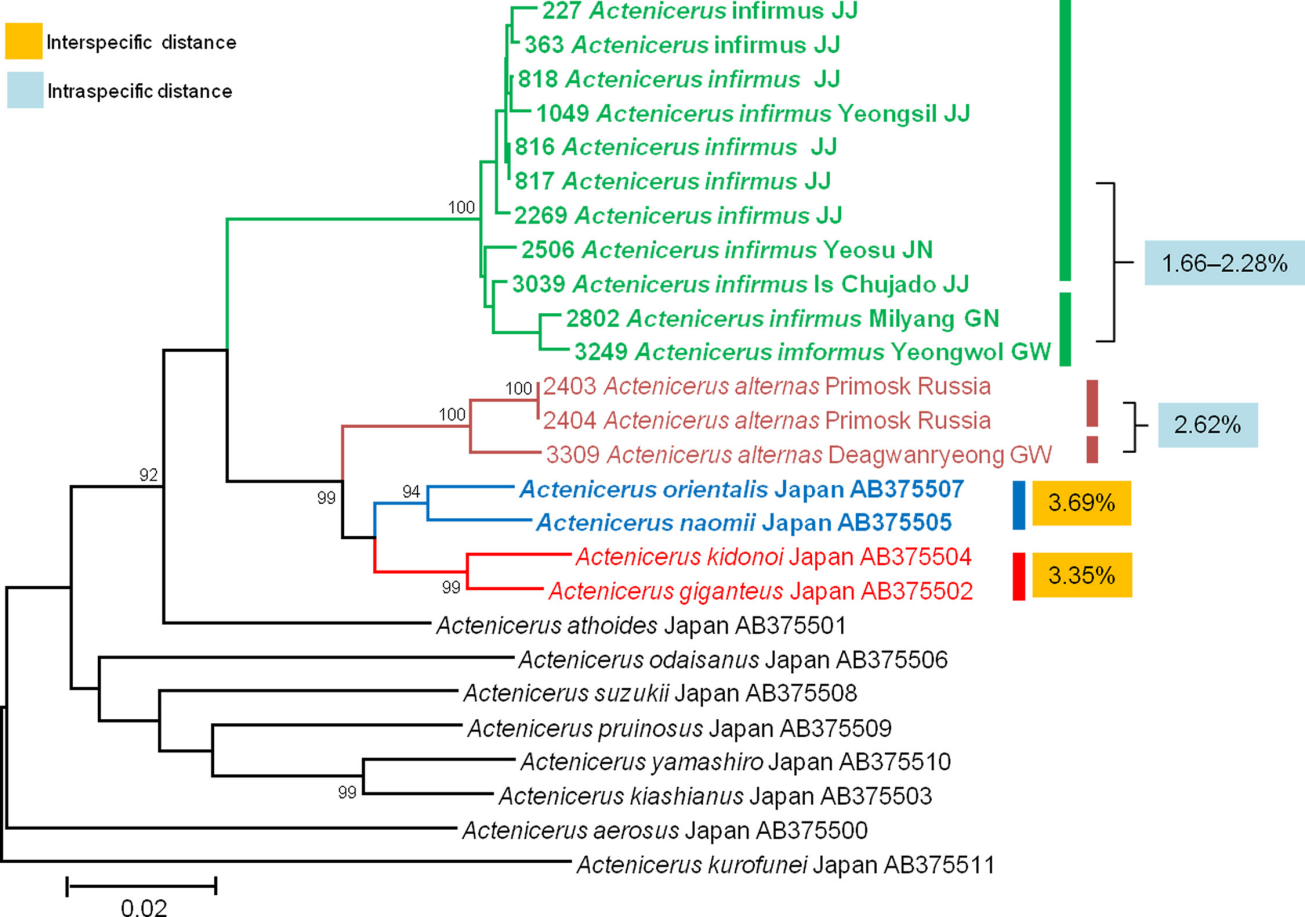
Neighbor-joining cladogram inferred from *COI* partial gene sequences of *Actenicerus* spp.

In contrast, members of the other 2 species pairs (*A*. *kidonoi*–*A*. *giganteus* and *Hemicrepidius* sp. 1–*H*. *oblongus*) could not be distinguished from one another in the DNA barcode approach using the 3.6% threshold. *A*. *giganteus* was originally identified by morphological differentiation from closely related species [74–76]; Ôhira subsequently identified and described *A*. *kidonoi* as the species closest to *A*. *giganteus* [77]. The boundary between these 2 species was also supported by our ABGD and PTP analyses. But the interspecific divergence between this species pair was 3.35% (**Fig 10**). The pair produced 2 MOTUs using the 2.5% threshold and in PTP analysis but only 1 MOTU using the 3.5% threshold.

The results from analyzing the species pair *H*. sp. 1–*H*. *oblongus* were similar to those of *A*. *kidonoi*–*A*. *giganteus* when applying 3.6% threshold value (**Table 2**) and both *X* values in ABGD (**Table 4**). The first author examined a male specimen and found a candidate new species, *H*. sp. 1. This putative morphospecies is extremely similar to *H*. *oblongus* but can be easily distinguished from the latter by a more sinuate base of the hind angle, a globular 2nd antennomere, and a 3rd antennomere longer than the 2nd. *H*. sp. 1 was closest to the Korean population of *H*. *oblongus* using interspecific genetic distance (range: 2.74%–3.37%, **S3 Table**) but was clustered as sister to a pseudocryptic species (no. 2397) detected from *H*. *oblongus* (**Fig 6**). Although this is a special case of clearer species delineation in morphology than by DNA barcoding, the interspecific distances overlapped with the intraspecific distances (range: 3.23%–3.66%) of four species (*A*. *pucticollis*, *H*. *oblongus*, *H*. *seccessus*, and Ctenicerini gen. and sp. 1) and two distinct lineages of *S*. *coreanus*. Inter- and intraspecific boundaries were difficult to detect in DNA barcoding for these examined species, suggesting that each taxon has evolved differently. Further studies using more specimens and sensitive genetic markers are needed.

#### 5) Low genetic divergences between morphologically determined subspecies

**Fig 11:**
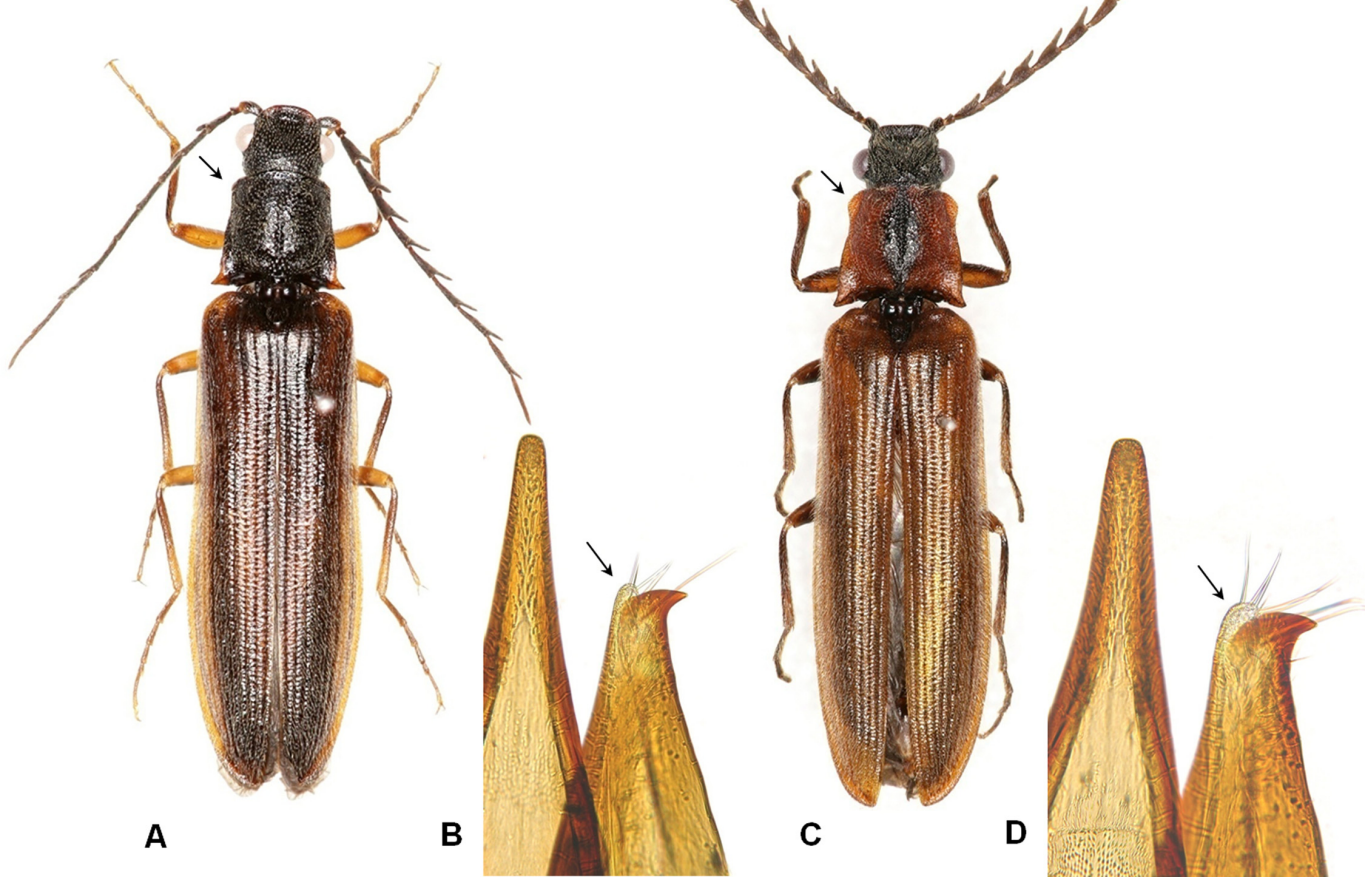
Two subspecies of *Denticollis nigricollis*. A and B: *D*. *n*. *nigricollis*; C and D: *D*. *n*. subsp. 1 (a candidate new subspecies identified by morphological examination). A and C: Dorsal view of adult; B and D: Aedeagus.

**Fig 12:**
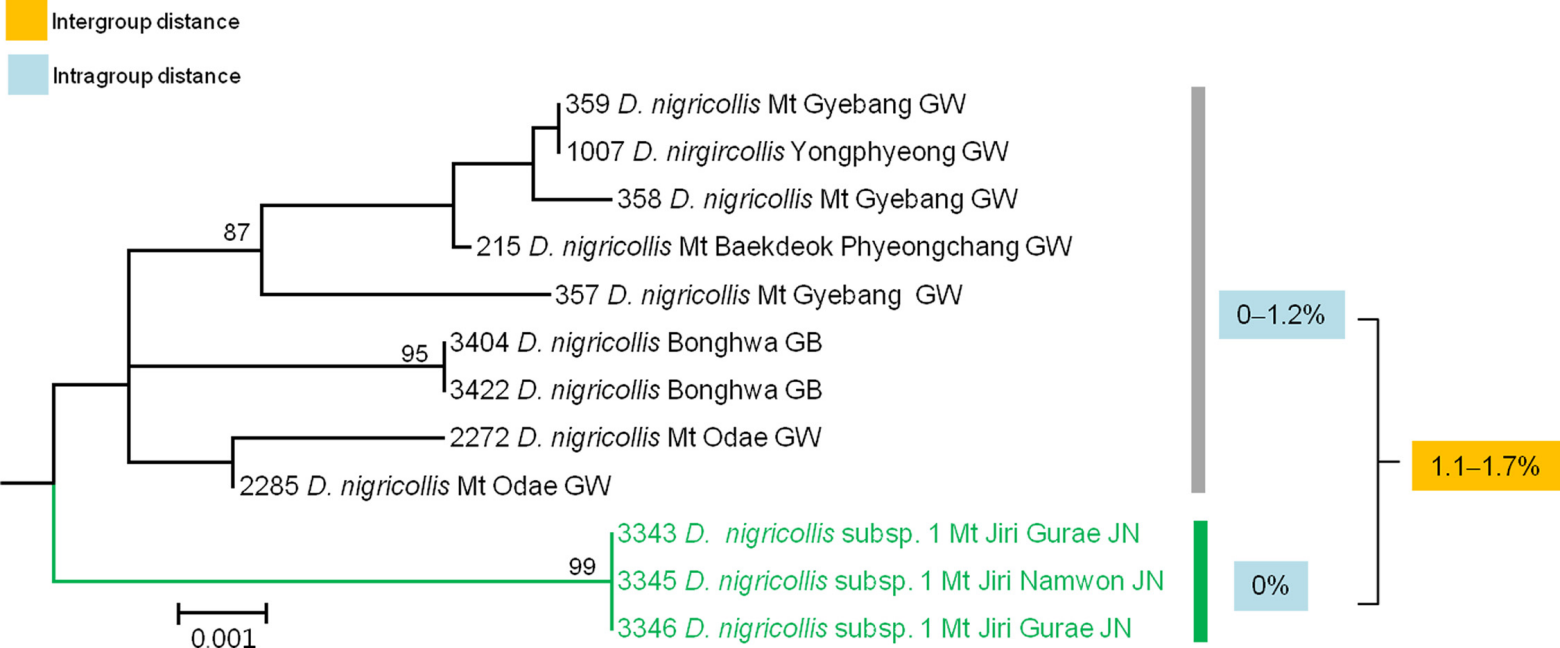
Neighbor-joining cladogram inferred from *COI* partial gene sequences of *Denticollis nigricollis*.

Within *Denticollis nigricollis*, we found a geographically separated population in Mount Jiri, located in south part of Korea that differed morphologically from other Korean populations. The first author identified this population as a candidate new subspecies based on several morphological differences and its geographical isolation. The previously identified nominotypical subspecies *D*. *n*. *nigricollis* has a normal anterior corner (**Fig 11A**) at the pronotal lateral margin and a narrowly shaped apex of the aedeagal paramere (**Fig 11B**), whereas *D*. *n*. subsp. 1 has an expanded anterior corner (**Fig 11C**) at the pronotal lateral margin and a more widely shaped apex of the paramere (**Fig 11D**). The differentiation of the aedeagus between the two subspecies is subtle and may be the result of interbreeding. Analysis of the *COI* sequences of these two subspecies revealed unexpectedly low genetic divergences (range: 1.1%–1.7%), but they formed two discrete clades (**Fig 12**). *D*. *n*. *nigricollis* consisted of nine individuals in Clade-A represented as monophyletic with low genetic divergences (range: 0–1.20%). *D*. *n*. subsp. 1 consisted of three test samples in Clade-B clustered into a group without genetic divergence. The discrepancies between the results of morphological examination and DNA barcoding make delimitating these groups into subspecies difficult, particularly because we detected no subspecies in 11 distinct species with varying intraspecific distances ranging from 0% to 3.67%, except for *H*. *seccessus* (**Table 3**). We hypothesize that evolution of two subspecies may signify incipient speciation as an adaption to locally distinct environments. Previous research has also suggested this phenomenon in *A*. *vittatus* [25]. Additional molecular studies, such as using multiple genetic markers, are necessary to confirm our hypothesis.

#### 6) DNA barcode sharing between morphologically distinct species

**Fig 13:**
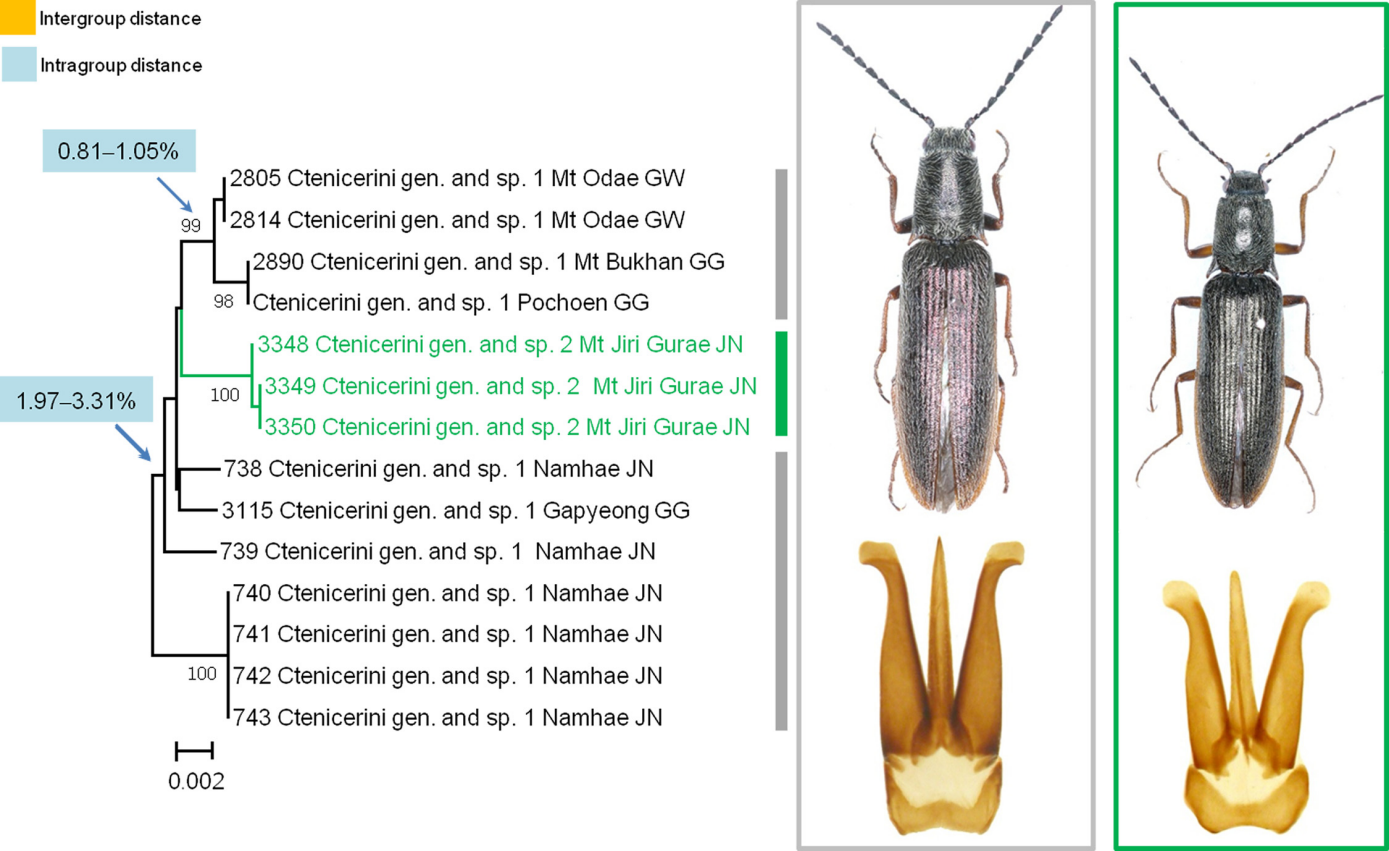
Neighbor-joining cladogram inferred from *COI* partial gene sequences of Ctenicerini gen. and sp. 1 and sp. 2.

Ctenicerini gen. and sp. 1 and sp. 2 were confirmed as two easily distinguishable morphospecies. Based on morphological analysis, we defined this Ctenicerini gen. putatively as a new genus with two new species. This candidate new taxon at the genus level is morphologically similar to the genus *Actenicerus* but can be easily distinguished by shorter hind angles of the pronotum, not laterally divergent with a vestige carina, and a nearly straight posterior margin of the hypomeron near the apex of the hind angle in ventral view. At the species level, these two candidate species are easily distinguished by different body shapes, sizes, and coloration and decisively distinguished by the shape of the aedeagal paramere apex (**Fig 13**). Given these differences, the identification of these two species cannot be “species oversplit” [38]. However, the DNA barcode result showed that these 2 putative species were part of the same group; Ctenicerini gen. and sp. 1 displayed paraphyly with Ctenicerini gen. and sp. 2 in NJ tree and PTP analyses (**Fig 13, S2 File**); and their ambiguous genetic distances (range: 2.12%–3.54%) produced a single MOTU in mothur using a 3.6% threshold and in ABGD analysis (**Table 4**), which made detecting a species boundary by genetic methods difficult. We first suspected superimposed substitutions, known as “multiple hits”, of the sequences between the two species and then additionally reexamined genetic distance using uncorrected measures such as the Jukes-Cantor model [78] in MEGA to compare with the Kimura 2–parameter values. However, the results of using this model were similar to the those of the other methods used. Therefore, we attributed the observed phenomenon in these two putative species to incomplete lineage sorting of ancestral mitochondrial DNA polymorphisms [79], introgression of mitochondrial DNA through interspecific hybridization [38, 80], or an affecting *Wolbachia* infection causing bias in the genetic structure of mitochondria [81]. This matter also requires additional analyses, including the use of multiple nuclear gene markers and morphological examination of more specimens in different life stages as well as consideration of ecological features to resolve the discrepancy we found between morphological examination and genetic analyses.

#### 7) Raising subspecific rank to specific rank by DNA barcoding result

Within the genus *Hemicrepidius*, *H*. *seccessus hallaensis* was recently described from specimens collected in Jeju Island, Korea by Kishii and Paik [42]. The examined *H*. *s*. *hallaensis* specimens have several diagnostic characteristics at the species level including more elongated antennae, apical two antennomeres extending beyond the apex of the hind angles, and different aedeagal shape when compared to other congeners. Furthermore, the DNA barcode result unequivocally showed that this subspecies is distinct from *H*. *seccessus* collected from Japan by having a large genetic distance (range: 6.1%–9.3%) and by clustering with *H*. sp. 2 (**Fig 6**). Therefore, we propose that this subspecies be elevated to species, *H*. *hallaensis* **stat. nov.**

## Discussion

In this study, we investigated the existence of cryptic and/or pseudocryptic species in 84 morphospecies within the Denticollid subfamily. Using DNA barcoding, we uncovered 6 hidden species: 5 hidden species from *A*. *vittatus*, *S*. *coreanus*, *S*. *koryeoensis*, *H*. *oblongus*, and *Stenagostus umbratilis* (intraspecific genetic distances of >5.0%) and a hidden species in *P*. *aurichalceum* using a 3.6% threshold value and re-examination of morphological features. Our findings suggest that, for these hidden species, divergence occurred through fast genetic changes despite subtle to no change in morphological features. On the other hand, we found two instances of distinguishable morphology but ambiguous sequence divergences in Ctenicerini gen. sp. 1 and C. sp. 2 and in the two subspecies of *Denticollis nigricollis*. This DNA barcode sharing indicates that their morphological differentiations are occurring more rapidly than their *COI* gene divergence. From this perspective, the morphologically separated species pairs with the relatively low interspecific genetic divergences (e.g., *H*. sp. 1–*H*. *oblongu*s and *A*. *giganteus*–*A*. *kidonoi*) are considered to have undergone incomplete lineage sorting, introgression, or recent speciation in the *COI* gene; however, their morphological differences are sufficient to confirm speciation. More studies examining other nuclear loci are needed to reveal their exact evolutionary pathway.

**Fig 14:**
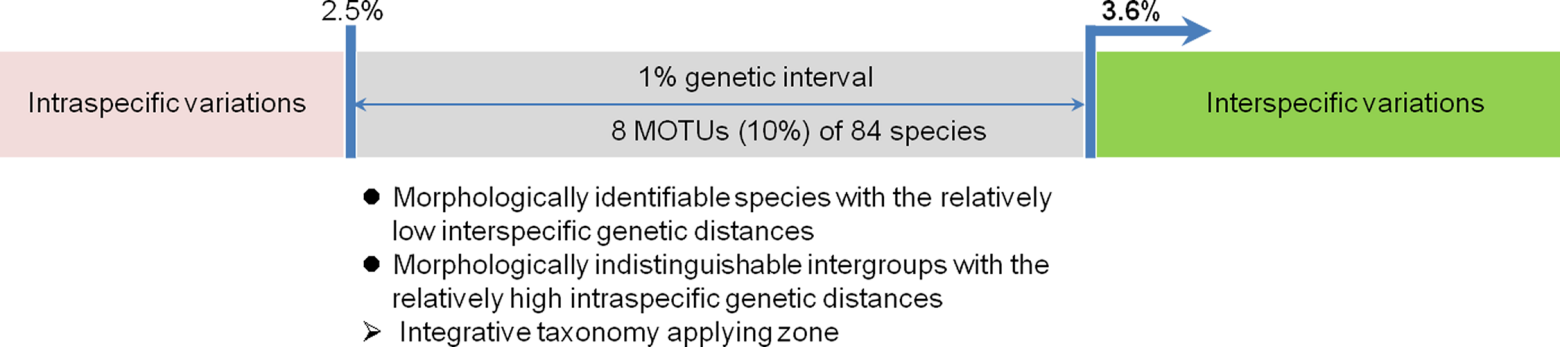
Schematic definition for a suitable threshold value (3.6%) for genetic distances and zone requiring integrative taxonomy (2.5–3.5%) suggested for the Denticollid taxa.

A suitable threshold value for insect species identification is typically between 2% and 5% but is different in each group: for example, a 2% threshold value is appropriate in several insect orders, such as Ephemeroptera [82], Lepidoptera [83, 84], Hymenoptera [85], Plecoptera, and Trichoptera [82, 86]; a 2.2% threshold value is used in the true bugs of Heteroptera [87, 88]; a 2.5% threshold value is used in Dytiscidae, Hydrophilidae, and Scarabaeidae of Coleoptera [89]; and thresholds between 3% and 5% are used in several dipteran taxa [90–93]. From our investigations, we determined that a conservative 3.6% threshold value applied to our DNA barcode reference library was suitable for delimitating species within Denticollinae. Applying this threshold to genetic analysis of our original 84 morphospecies, we identified 87 MOTUs, which uncovered 7 hidden species (including one in *A*. *puncticollis*) and eliminated 4 species. This result was similar to the 89 MOTUs identified by ABGD analysis. Notably, the MOTUs identified in the interval from 2.5% to 3.5% contained 3 morphologically identifiable species (**Table 2**) and morphologically indistinguishable subgroups in 10 single species, excluding *A*. *puncticollis*, whose morphological features were not examined directly in this study (**Table 3**). The species boundaries for 3 species pairs could not be delimited solely by DNA barcoding approaches, identifying the risk of underestimating species number by molecular species identification without morphological examination. In contrast, adopting an inadequate threshold value, such as 2.5%, could mistakenly result in identifying distinct MOTUs for subgroups within species, such as was the case with the 10 species mentioned above. Such an inappropriate threshold would then result in an overestimation of species richness in given taxonomic groups. In this study, we found 8 MOTUs in the 2.5% to 3.5% threshold range, even though this 1% genetic interval was a very small difference compared with the whole interspecific genetic distance range. However, this is considered a very important genetic range in species delimitation when used with integrative taxonomic information such as morphological, behavioral, and ecological differences [17, 94] (**Fig 14**). In our case, prior knowledge of the morphological differences already recognized for the 8 MOTUs contributed to the decision of whether the ambiguous genetic divergences should be considered as incomplete lineage sorting or simply intraspecific genetic variation. However, these assumptions have to be supported by additional hypotheses testing. Notably, the *COI* gene is not the fastest evolving gene among the 13 mitochondrial protein coding genes in insects. For example, seven mitochondrial genes (*ATP6*, *COII*, *COIII*, *ND2*, *ND4L*, *ND5*, and *ND6*) evolve divergences faster than *COI* in Hemiptera and Lepidoptera [95, 96], and several nuclear protein coding genes, such as *CAD* and *DDC*, have similar or faster divergence rates than *COI* in butterfly species [97]. Our empirical experiences with some mitochondrial protein coding genes (*ND1–5*, *COII*, and *COIII*) and nuclear protein coding genes (*CAD* and *DDC*) also revealed faster divergence rates in genes other than *COI* in certain butterflies and locusts of Orthoptera (Han, unpublished data). These genes may be sites that provide more useful information with which to distinguish species pairs that have low genetic distances in *COI* analysis. Elias et al. [98] pointed out the dangers of relying solely on mtDNA data to define species with polymorphism markers in the genus *Mechanitis* of Nymphalidae in Lepidoptera. However, we consider this caution to be applicable only to the relatively few taxa demonstrating problematic species delimitation throughout their DNA barcode library.

Our study also found geographically correlated intraspecific divergences in several species, such as the *H*. *coreanus* population collected from Jeju Island (**Fig 6**), the Russian and Korean populations of *H*. *oblongus* (nos. 3069 and 3103), the Jeju population of *Corymbitodes* sp. 1 (no. 2303), and the Jeju populations of *A*. *infirmus*. These may not necessarily represent cryptic species or subspecific taxonomic units. However, based on the current data and given the small interspecific distances involved, further sampling is needed to reveal more reliable species identification. These cases also highlight the importance of comprehensive sampling across different populations and geographic regions. Notably, DNA barcode sharing represented in Ctenicerini gen. and sp. 1 and sp. 2 is a case of mtDNA paraphyly, which may be caused by introgression [38, 80, 81, 99] or incomplete lineage sorting in recent speciation events [79].

This study shows that DNA barcoding is very helpful to identify the taxonomically difficult species with subtle morphological characteristics in Denticollinae. Furthermore, most studies of nominal elaterid species have been focused on adult-based identification even though the larval stage is more affected by ecological damage in agricultural and forest systems. Relatively few larval studies have been carried out by few elaterid specialists [29, 31, 100–102]. Our DNA barcode reference library will also provide helpful information for larval species identification. Therefore, we encourage additional DNA barcode studies for polymorphic species, polytypic species occurring in sympatric and allopatric populations, and single species that have an extensively large habitat.

## Supporting Information

S1 File 421 *COI* sequences (658 bp) of 84 species belonging to the subfamily Denticollinae. (FAS)

S2 File Maximum likelihood tree-based on the PTP model. (PDF)

S1 Table List of 421 *COI* sequences of 84 species belonging to 36 genera and three tribes within the subfamily Denticollinae. (XLSX)

S2 Table Intraspecific genetic distances within morphospecies and detection of the separated groups with large genetic distances in Denticollinae (XLSX)

S3 Table Intraspecific and interspecific genetic distances within and between groups or species represented by low and ambiguous genetic distances. (XLSX)
