# Supplementary figures and images for: Reassessment of Species Diversity of the Subfamily Denticollinae (Coleoptera: Elateridae) through DNA Barcoding
Source: PLoS One. 2016 Feb 5;11(2):e0148602. doi: 10.1371/journal.pone.0148602 (PMC4744053; doi:10.1371/journal.pone.0148602)

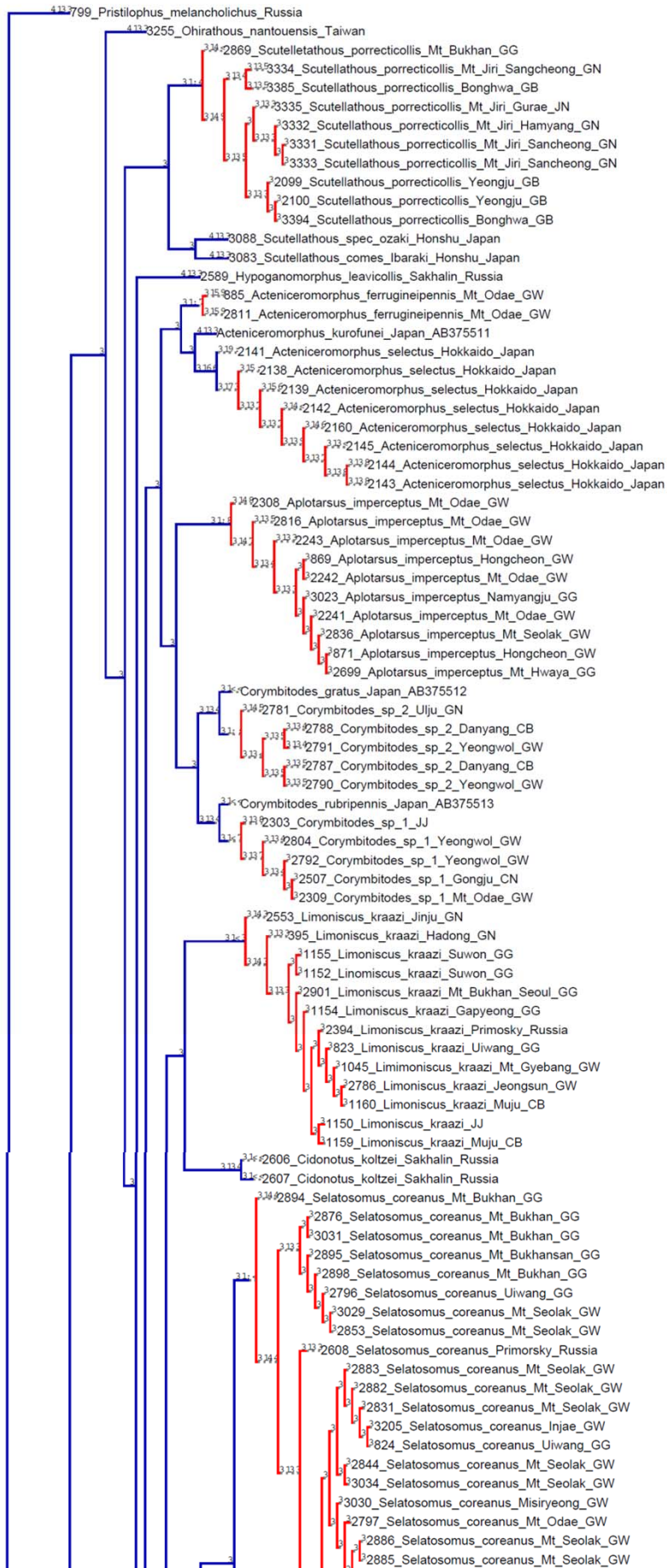

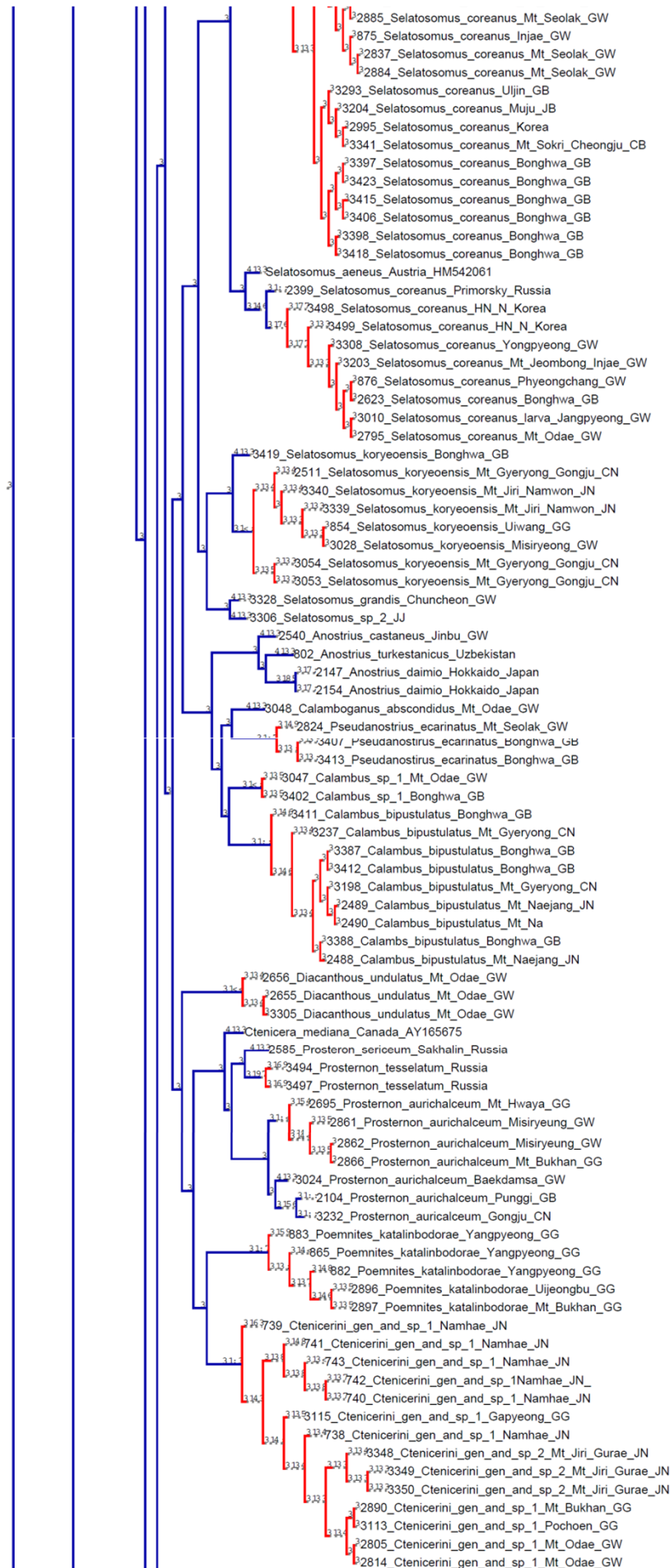

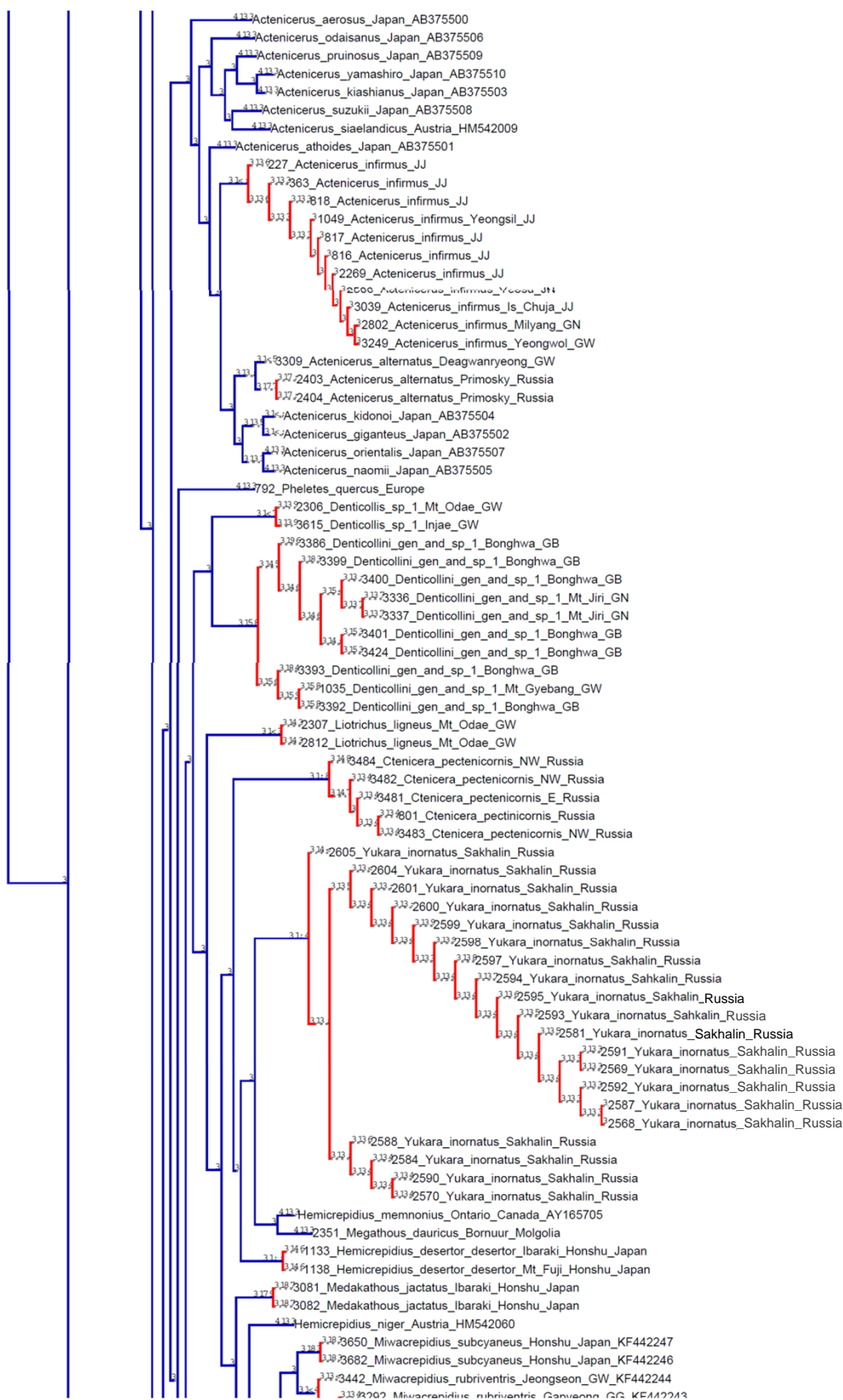

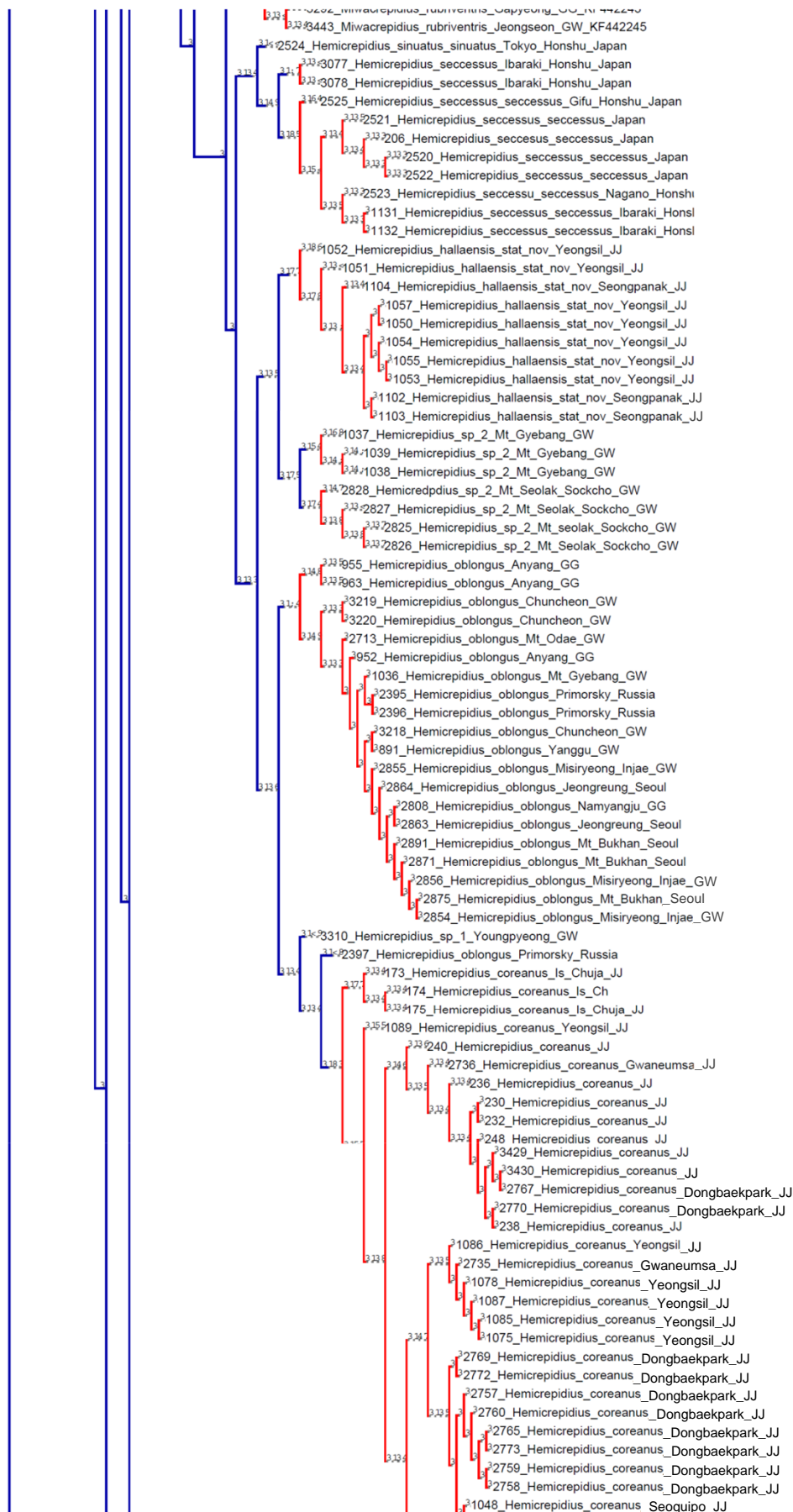

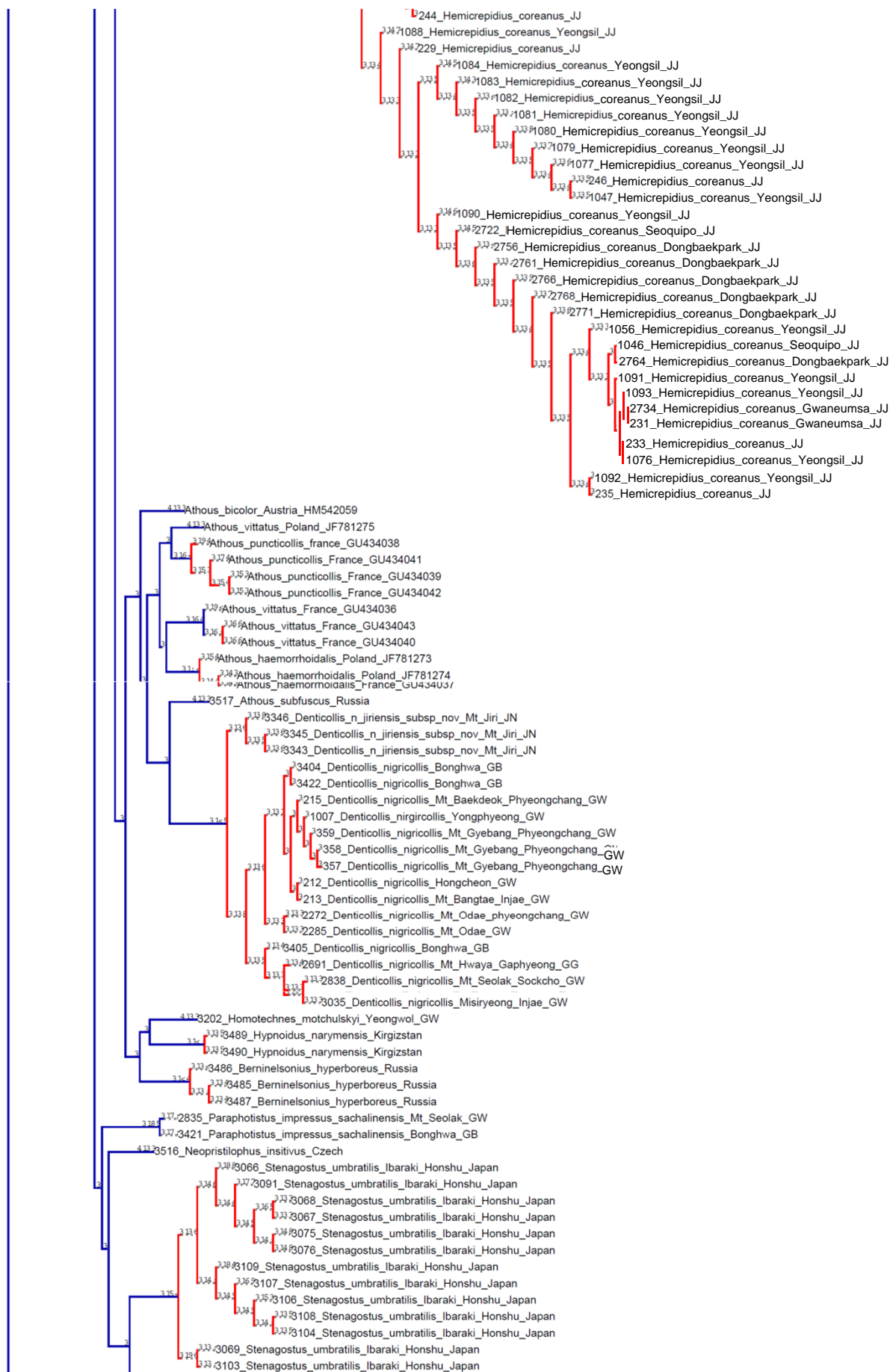

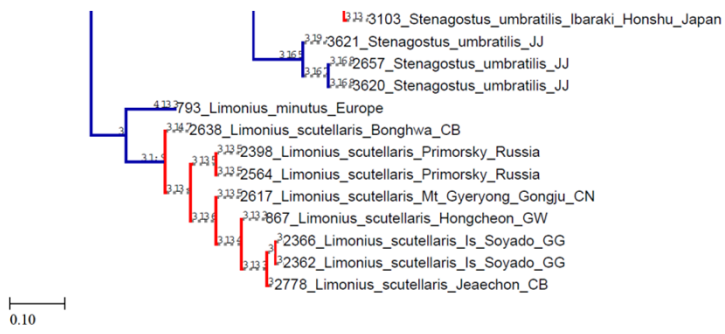

Supplement: S2 File — (PDF) [file pone.0148602.s002.pdf]
